# Combined effects of nitrogen dioxide and ozone air pollution on maternal liver function during pregnancy: a birth cohort study in China

**DOI:** 10.1265/ehpm.26-00057

**Published:** 2026-06-12

**Authors:** Zhijian Chen, Zhongai Ouyang, Weigui Ni, Bingyi Lin, Haoqu Zheng, Long Jiang, Bo Wu, Lijuan Lai, Minting Zhu, Yi Jing, Zhuang Liu, Xi Yu, Jingjie Fan

**Affiliations:** 1 Faculty of Medicine, Macau University of Science and Technology, Avenida Wai Long, Taipa, Macau, China; 2 Department of Preventive Healthcare, Shenzhen Maternity and Child Healthcare Hospital, Women and Children’s Medical Center, Southern Medical University, Shenzhen 518028, China; 3 School of Public Health, Southern Medical University, Guangzhou 510515, China; 4 Department of Dermatology, Shenzhen Maternity and Child Healthcare Hospital, Women and Children’s Medical Center, Southern Medical University, Shenzhen 518028, China

**Keywords:** Nitrogen dioxide (NO_2_), Ozone (O_3_), Air pollution, Liver function, Pregnant woman, Combined effects

## Abstract

**Background:**

Nitrogen dioxide (NO_2_) and ozone (O_3_) exposure is associated with alterations in liver function, but epidemiological evidence in pregnant women remains limited, especially regarding the combined effects. This study aimed to investigate the associations of exposure to NO_2_ and O_3_ with maternal liver function and their roles in the combined effect of air pollutant mixture.

**Methods:**

A birth cohort study with 11,909 pregnant women was conducted in Shenzhen, China. Exposure concentration of air pollutants during the first trimester were assessed based on the residential addresses of the pregnant women. Generalized additive model was used to analyze the association of NO_2_ and O_3_ exposure with maternal liver function. Furthermore, quantile G-computation model was used to investigate the combined effects of air pollutant mixture and the contribution of each pollutant.

**Results:**

Each 10 µg/m^3^ increase in first-trimester NO_2_ exposure was associated with elevated alanine aminotransferase (ALT) and total bilirubin (TBIL) in both the second trimester (ALT: 15.45%, 95% CI: 10.12%, 21.04%; TBIL: 6.69%, 95% CI: 3.75%, 9.72%) and third trimester (ALT: 3.89%, 95% CI: 0.32%, 7.59%; TBIL: 9.27%, 95% CI: 6.52%, 12.10%). For O_3_, first-trimester exposure showed a positive association with second-trimester TBIL (2.03%, 95% CI: 1.25%, 2.81%), but was inversely associated with third-trimester ALT (−2.73%, 95% CI: −3.86%, −1.58%) and TBIL (−0.81%, 95% CI: −1.45%, −0.17%). These associations remained significant in the two-pollutant model. The Qgcomp analysis revealed that a one-quartile increase in the air pollutant mixture was associated with a 2.05% (95% CI: 0.56%, 3.56%) increase in third-trimester TBIL, with NO_2_ accounting for 55.6% of the positive weight in the model.

**Conclusions:**

First-trimester NO_2_ exposure was associated with elevated maternal liver function biomarkers in both the second and third trimesters, while O_3_ showed trimester-dependent associations. Exposure to air pollutant mixture was associated with increased third-trimester TBIL, and NO_2_ may be the predominant relative contributor. These findings highlight the need for further studies and public health attention to air pollution exposure during pregnancy to protect maternal and fetal health.

**Supplementary information:**

The online version contains supplementary material available at https://doi.org/10.1265/ehpm.26-00057.

## Graphical Abstract

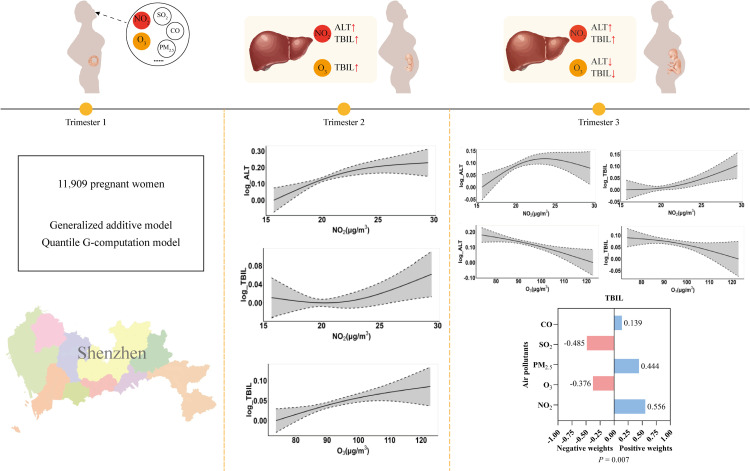

## 1. Introduction

Pregnant women represent a population of significant societal concern, as their health status directly impacts both maternal well-being and fetal development. During pregnancy, the maternal body undergoes profound physiological and metabolic adaptations [1, 2], with the liver bearing a substantial metabolic burden in supporting fetal growth, hormone clearance, and detoxification processes [3, 4]. Consequently, liver function serves as a critical indicator for evaluating maternal health and pregnancy outcomes. Studies have demonstrated that impaired liver function is closely associated with elevated risks of gestational diabetes mellitus, preeclampsia, and preterm birth [5–7]. Therefore, maintaining stable liver function during this critical susceptible period is critically important for ensuring maternal health and promoting optimal fetal development.

Among various environmental exposure factors, ambient air pollution poses a substantial threat to maternal and fetal health. Within complex pollutant mixtures, gaseous pollutants are particularly concerning due to their high reactivity and potential to induce systemic oxidative stress. Nitrogen dioxide (NO_2_) and ozone (O_3_), as key oxidative gaseous pollutants [8], are widely distributed in the atmospheric environment and exert well-established systemic toxicity [9–13]. Given that these two pollutants often coexist in real-world environments, current research increasingly emphasizes that their co-exposure may exert complex combined effects on human health [14, 15]. Although strict pollution control policies have reduced atmospheric pollutant concentrations across China and many other countries in recent years [16], China still exhibits a distinctive combined pollution pattern, characterized by persistently high NO_2_ levels and sustained increases in O_3_ concentrations [17, 18]. Global long-term monitoring data (2000–2019) demonstrate that the annual average NO_2_ concentration in China is significantly higher than that in Europe, the United States, and India [18]. With regard to O_3_ pollution, China, together with India, Mexico, the Republic of Korea, and Japan, is among the regions with the most frequent O_3_ exceedances worldwide [19], and O_3_ exposure levels in urban areas have shown a sustained upward trend [20]. Consequently, the high-level and persistent co-exposure burden of NO_2_ and O_3_ represents a major environmental health challenge in China.

Numerous epidemiological studies have shown that ambient exposure to NO_2_ and O_3_ are closely associated with alterations in liver function. For instance, a large population-based longitudinal study of older adults observed that both NO_2_ and O_3_ exposure were significantly associated with elevated liver enzymes, including aspartate aminotransferase (AST) and alanine aminotransferase (ALT) [21]. Another study focusing on patients with schizophrenia demonstrated that long-term exposure to NO_2_ significantly increased levels of ALT and γ-glutamyl transferase (GGT) in these individuals [22]. Nevertheless, epidemiological evidence regarding the associations of NO_2_ and O_3_ exposure with maternal liver function remains extremely limited. A recent birth cohort study performed in Foshan, southern China, preliminarily indicated that prenatal O_3_ exposure was positively associated with ALT levels during the third trimester of pregnancy, whereas no significant association was observed for NO_2_ exposure [23]. However, this study only assessed liver function at a single time window and focused on the independent effects of individual pollutants, which may not fully capture the temporal characteristics of prenatal exposure or account for the context of co-exposure to multiple pollutants. In fact, in real-world environments, the environmental exposure of the population in daily life is not the independent effect of a single pollutant but rather the combined effects of multiple pollutants that jointly drive health outcomes [24]. However, the current lack of research on the joint associations between air pollutant mixture and maternal liver function represents a critical knowledge gap. It is essential to clarify the roles of NO_2_ and O_3_ in the associations between air pollutant exposure and liver function in pregnant women within the context of multi-pollutant co-exposure.

Given the heavy burden of combined NO_2_ and O_3_ pollution in China, the high susceptibility of pregnant women to environmental hazards, and the existing critical research gaps in this field, it is imperative to conduct targeted epidemiological research among Chinese pregnant populations. Based on a retrospective birth cohort, this study systematically investigated the associations of exposure to NO_2_ and O_3_ with maternal liver function in pregnant women, and further explored their contributions to the combined association of air pollutant mixtures with maternal liver function. We aim to provide epidemiological evidence for the formulation of environmental health policies and maternal-fetal health care.

## 2. Methods

### 2.1. Study population

Participants in this research were sourced from a pregnant population enrolled in the birth cohort study conducted by Shenzhen Maternity and Child Healthcare Hospital. Data were collected from a total of 25,208 pregnant women who registered at the hospital and had delivery outcomes between July 2021 and March 2023. Pregnant women were initially enrolled in the study if they fulfilled the following criteria: (1) aged 18 and above; (2) underwent liver function tests during the prenatal visits in both the second and third trimesters; (3) submitted their residential address; (4) delivered at the Hospital. Individuals with multiple births (N = 497), a history of hepatitis (N = 708), and missing critical variable details such as NO_2_ exposure levels (N = 58), O_3_ exposure levels (N = 149), mean temperature and relative humidity (N = 835), as well as physical burden (N = 1) were excluded. Ultimately, 11,909 participants were included in the study. Figure S1 illustrates the process of participant inclusion and exclusion. Table S1 shows that most baseline variables were not statistically different between included and excluded participants, and the baseline characteristics were generally balanced between the two groups, indicating no significant selection bias. Each participant completed a detailed questionnaire (Table S2) during the registration and enrollment process. The contents of the questionnaire were demographic characteristics, residential address, life behaviors, medication history, and medical history. Information on pregnancy conditions, pregnancy complications, and liver function test results were obtained from the clinical information system and laboratory system.

In this study, the first trimester was defined as the period from conception to 13 weeks of gestation, the second trimester as 14 to 27 weeks of gestation, and the third trimester as 28 weeks of gestation until delivery.

### 2.2. Liver function assessment

Fasting blood samples were collected from each pregnant woman using a pro-coagulation tube during their second and third trimesters. The samples were then centrifuged at 3500 rpm for 10 minutes, and the serum was separated. Malate dehydrogenase, lactate dehydrogenase, and diazotization methods were employed to measure AST, ALT, and total bilirubin (TBIL) levels, respectively, by certified clinical laboratory personnel with a Beckman Coulter biochemical analyzer (California, USA), a classic automated biochemical detection system renowned for its high automation and reliability, which is widely used in tertiary hospitals across China [25]. All clinical laboratory personnel involved in the clinical testing passed strict professional assessments and held valid qualification certificates. All operations were carried out in strict accordance with standard clinical laboratory operating procedures, including standardized sample processing, instrument calibration, and internal quality control, to ensure the accuracy and repeatability of liver function test results. According to the reference ranges of liver enzymes in the laboratory and relevant references, an ALT or AST level exceeding 40 U/L is considered an abnormally elevated liver enzyme [23].

### 2.3. Air pollution exposure assessment

The exposure levels of NO_2_ and O_3_ were derived from the ChinaHighNO_2_ and ChinaHighO_3_ dataset, which is part of the ChinaHighAirPollutants (CHAP) series. Utilizing artificial intelligence, this dataset synthesizes ground monitoring, satellite remote sensing, atmospheric reanalysis, and modeling data, explicitly addressing the spatiotemporal dynamics of air pollution. The model exhibits a cross-validation coefficient of determination (CV-R^2^) of 0.93 and 0.87 for NO_2_ and O_3_, with a spatial resolution of 1 km [26–28]. Based on this high quality dataset, we collected daily NO_2_ and O_3_ exposure concentrations for each participant. Finally, we assessed the mean levels of exposure to air pollutants for each participant in the first trimester.

The data of daily mean temperature were sourced from the ERA5 reanalysis provided by the European Centre for Medium-Range Weather Forecasts (ECMWF). The relative humidity values were derived from an analysis of the 2 m dew point and air temperature data in the dataset [29, 30]. The data for relative humidity and mean temperature were derived from the grid corresponding to the residential addresses of each participant, and the exposure levels of relative humidity and mean temperature were calculated.

### 2.4. Statistical analysis

Mean ± SD or number (%) were used to describe the participant characteristics. The detailed statistical analysis of our study was as follows:

Firstly, to examine the associations between NO_2_ and O_3_ exposures during the first trimester and maternal liver function, the Generalized Additive Model (GAM) was utilized. The concentrations of liver function biomarkers underwent natural logarithm transformation to make them conform to a normal distribution. The exposure levels of air pollutants were either treated as categorized into quartiles or continuous variables for model construction. For trend analysis, the calculation of the *P*-value in the model relied on the strategy of treating the median of each quartile as a continuous variable. The findings were displayed as the percentage variation in the biomarkers of liver function [exp(*β*) − 1 × 100%] and the 95% confidence interval (CI) corresponding to an increase of 10 µg/m^3^ in air pollutants. To further evaluate the dose-response relationships between NO_2_, O_3_ and maternal liver function, a natural cubic spline function was utilized. Drawing on previous research experience and considering the Root Mean Square Error value from the Generalized Cross Validation [31, 32], the degree of freedom of the function was determined to be three (*df* = 3). Using a spline function, adjustments to the crude model were made only for relative humidity and mean temperature (*df* = 3). The single-pollutant model further adjusted for covariates and confounding factors including maternal age, gestational age at liver function testing, pregnancy methods, pre-pregnancy BMI, education level, smoking status, alcohol-drinking status, gestational diabetes, gestational hypertension, status of HBsAg, physical burden, taking medication, and season at liver function testing [33–36]. The two-pollutant model was constructed by additionally incorporating either NO_2_ or O_3_ to examine the joint effects.

Secondly, the association between air pollutants and the risk of abnormally elevated liver function was analyzed using the Generalized Additive Model with logistic regression. The aforementioned covariates and confounding factors were adjusted for in the model. The results were expressed as the odds ratio (OR) and its 95% CI for per 10 µg/m^3^ increase in either NO_2_ or O_3_.

Thirdly, to explore the potential combined association of air pollutant mixture with maternal liver function and the relative contributions of NO_2_ and O_3_ to these combined effects, the quantile G-computation model was applied. The quantile G-computation model is a method further improved based on the weighted quantile sum regression. The model allows pollutants to exhibit complex interactions beyond simple linear effects and does not require the assumption of directional homogeneity, meaning that the effects of each pollutant can be in different directions. Additionally, the correlations among different pollutants have little impact on the estimation of the overall effect. This high robustness to multicollinearity effectively avoids the instability of parameter estimates commonly caused by collinearity in traditional regression models [37, 38]. We included the first-trimester exposure concentrations of common air pollutants such as NO_2_, O_3_, particulate matter with an aerodynamic diameter of ≤2.5 µm (PM_2.5_), sulfur dioxide (SO_2_) and carbon monoxide (CO) in the mixed-pollutant model. The estimated value (*ψ*) and the coefficient (*β*) of each pollutant were subsequently derived. An assessment was then conducted on the combined effect of the air pollutant mixture.

Fourthly, a stratified analysis was performed by incorporating a multiplicative interaction term between NO_2_/O_3_ and each covariate to explore the influence of these covariates on the association between air pollution exposure and liver function. The covariates included pregnancy methods, pre-pregnancy BMI, maternal age, education level, alcohol-drinking status, gestational diabetes, gestational hypertension, and status of HBsAg. Stratified analysis was not performed for smoking status due to substantial sample size discrepancies across its subgroups.

R software (R 4.3.3) was used for statistical analysis, and the involved packages include car, caret, Epi, ggplot2, gridExtra, lubridate, mgcv, plyr, psych, qgcomp, and splines. Statistical significance was defined as a *P* value ≤ 0.05 (two-tailed).

### 2.5. Sensitivity analysis

A comprehensive set of sensitivity analyses was performed to assess the robustness of the results in our study. Firstly, the *df* of the spline function was changed from 3 to 7 to examine the robustness of the effects of NO_2_ and O_3_. Secondly, to reduce potential confounding effects of comorbidities, participants with gestational hypertension, gestational diabetes, or either of these conditions were excluded. Thirdly, to confirm the relatively short-term effects of NO_2_ and O_3_, we further examined the associations of second-trimester NO_2_ and O_3_ exposures with third-trimester maternal liver function. Fourthly, considering the cumulative exposure effect of air pollutants during the second and third trimesters on maternal liver function, we constructed a two-period model. This model was adjusted for the exposure concentration from the second trimester alone, as well as the average exposure concentrations across both the second and third trimesters, to explore the independent effect of first-trimester exposure.

## 3. Results

### 3.1. Descriptive statistics

Table 1 shows the general characteristics of 11,909 participants. The mean maternal age of the study subjects was 31.45 years (SD = 4.06 years). Liver function tests were performed at a mean gestational age of 24.96 ± 1.25 weeks (second trimester) and 34.72 ± 1.00 weeks (third trimester). The mean pre-pregnancy BMI level of these pregnant participants was 21.02 kg/m^2^ (SD = 2.73 kg/m^2^). Among these pregnant women, more than 90% achieved natural conception, and the vast majority had no smoking history (97.96%) or did not consume alcohol (90.94%). About 86% of the subjects had an undergraduate education or below. During the pregnancy of the subjects, 576 (4.84%) were diagnosed with gestational hypertension, 2759 (23.17%) were diagnosed with gestational diabetes, 340 (2.85%) were tested positive for HBsAg, and 38.62% of the subjects took medication during the first trimester.

**Table 1:** General characteristics of the pregnant women (N = 11,909)

**Characteristics**	**Mean ± SD or N (%)**
Maternal age (years)	31.45 ± 4.06
Pre-pregnancy BMI (kg/m^2^)	21.02 ± 2.73
Gestational age at liver function testing (weeks)	
Second trimester	24.96 ± 1.25
Third trimester	34.72 ± 1.00
Pregnancy methods	
Natural conception	10834 (90.97)
Assisted reproduction	1075 (9.03)
Education	
Below undergraduate	4865 (40.85)
Undergraduate	5413 (45.45)
Above undergraduate	1631 (13.70)
Cigarette smoking	
Never	11666 (97.96)
Current	36 (0.30)
Former	207 (1.74)
Alcohol drinking	
No	10830 (90.94)
Yes	1079 (9.06)
Physical burden	
Light	7952 (66.77)
Medium	3909 (32.83)
Heavy	48 (0.40)
Gestational hypertension	
No	11333 (95.16)
Yes	576 (4.84)
Gestational diabetes	
No	9150 (76.83)
Yes	2759 (23.17)
Status of HBsAg	
Negative	11569 (97.15)
Positive	340 (2.85)
Taking medication during the first trimester	
No	7310 (61.38)
Yes	4599 (38.62)
Season at liver function testing	
Warm seasons (May. to Oct.)	
Second trimester	4815 (40.43)
Third trimester	6429 (53.98)
Cold seasons (Nov. to Apr.)	
Second trimester	7094 (59.57)
Third trimester	5480 (46.02)
Elevated liver enzymes (ALT or AST > 40 (U/L))	
Second trimester	
>40 (U/L)	588 (4.94)
≤40 (U/L)	11321 (95.06)
Third trimester	
>40 (U/L)	214 (1.80)
≤40 (U/L)	11695 (98.20)

The mean concentrations of NO_2_ was 21.08 µg/m^3^ (SD = 3.54 µg/m^3^), while that of O_3_ was 93.40 µg/m^3^ (SD = 11.98 µg/m^3^). There was a weak negative correlation between the exposure concentrations of NO_2_ and O_3_ (Pearson’s r = −0.069, *P* < 0.05), suggesting minimal collinearity and supporting the stability of the two-pollutant model estimates. The mean temperature was 22.28 °C (SD = 4.30 °C) and the relative humidity was 77.67% (SD = 5.99%). The mean concentrations of the biomarkers of liver function were: ALT 17.83 U/L (SD = 13.00), AST 19.39 U/L (SD = 7.20), and TBIL 7.23 µmol/L (SD = 2.71). Corresponding values in the third trimester were, ALT 13.36 U/L (SD = 11.83), AST 18.48 U/L (SD = 7.45), and TBIL 8.13 µmol/L (SD = 2.90) (Table 2).

**Table 2:** Descriptive statistics of air pollution exposure, meteorological variables and liver function biomarkers

**Variables**	**Mean (SD)**	**Median**	**Percentile**		

			**25th**	**50th**	**75th**
NO_2_ (µg/m^3^)	21.08 (3.54)	20.37	18.40	20.37	22.97
O_3_ (µg/m^3^)	93.40 (11.98)	91.23	84.85	91.23	99.19
PM_2.5_ (µg/m^3^)	20.71 (6.20)	20.55	15.94	20.55	24.84
SO_2_ (µg/m^3^)	5.60 (0.60)	5.67	4.95	5.67	6.14
CO (mg/m^3^)	0.61 (0.04)	0.62	0.57	0.62	0.64
Mean temperature (°C)	22.28 (4.30)	22.64	18.07	22.64	26.57
Relative humidity (%)	77.67 (5.99)	77.24	72.17	77.24	83.63
Liver function biomarkers					
Second trimester					
ALT (U/L)	17.83 (13.00)	14.00	11.00	14.00	20.00
AST (U/L)	19.39 (7.20)	18.00	15.00	18.00	21.00
TBIL (µmol/L)	7.23 (2.71)	6.70	5.60	6.70	8.20
Third trimester					
ALT (U/L)	13.36 (11.83)	11.00	9.00	11.00	15.00
AST (U/L)	18.48 (7.45)	17.00	15.00	17.00	20.00
TBIL (µmol/L)	8.13 (2.90)	7.50	6.30	7.50	9.30

Exposure to air pollutants and meteorological factors during the first trimester.

### 3.2. Associations of NO_2_ and O_3_ air pollution exposure with maternal liver function

As shown in Table 3, in the single-pollutant model, each 10 µg/m^3^ increase in first-trimester NO_2_ exposure was associated with a 15.45% (95% CI: 10.12%, 21.04%) increase in second-trimester ALT and a 6.69% (95% CI: 3.75%, 9.72%) increase in second-trimester TBIL. Similarly, each 10 µg/m^3^ increase in first-trimester NO_2_ exposure was associated with a 3.89% (95% CI: 0.32%, 7.59%) increase in third-trimester ALT and a 9.27% (95% CI: 6.52%, 12.10%) increase in third-trimester TBIL. Regarding O_3_, each 10 µg/m^3^ increase in first-trimester O_3_ exposure was associated with a 2.03% (95% CI: 1.25%, 2.81%) increase in second-trimester TBIL. In contrast, during the third trimester, each 10 µg/m^3^ increase in first-trimester O_3_ exposure was associated with a 2.73% (95% CI: −3.86%, −1.58%) decrease in ALT, and a 0.81% (95% CI: −1.45%, −0.17%) decrease in TBIL. In the two-pollutant model, each 10 µg/m^3^ increase in first-trimester NO_2_ and O_3_ exposures remained significantly associated with ALT and TBIL. The crude model demonstrated similar results (Table S3).

**Table 3:** The linear associations of NO_2_ and O_3_ air pollution exposure with maternal liver function

**Liver function** **biomarkers**	**Quartiles and** **continuous measure**	**Single-pollutant model**				**Two-pollutant model**			
	
		**NO_2_**		**O_3_**		**NO_2_**		**O_3_**	
			
		**%Change (95%CI)**	***P* for trend**	**%Change (95%CI)**	***P* for trend**	**%Change (95%CI)**	***P* for trend**	**%Change (95%CI)**	***P* for trend**
Second trimester									
ALT	Q1	Ref		Ref		Ref		Ref	
	Q2	2.76 (−0.10, 5.71)		1.75 (−0.77, 4.33)		3.13 (0.14, 6.21)^a^		−0.08 (−2.60, 2.50)	
	Q3	9.81 (5.70, 14.08)^c^		3.49 (0.67, 6.39)^a^		9.72 (5.61, 14.00)^c^		0.22 (−2.62, 3.14)	
	Q4	12.09 (7.26, 17.15)^c^	<0.001^c^	5.00 (0.62, 9.58)^a^	0.009^b^	12.10 (7.26, 17.15)^c^	<0.001^c^	1.02 (−2.41, 4.57)	0.566
	Per 10 µg/m^3^	15.45 (10.12, 21.04)^c^		−0.41 (−1.57, 0.76)		17.02 (11.34, 23.00)^c^		−1.13 (−2.49, 0.26)	
AST	Q1	Ref		Ref		Ref		Ref	
	Q2	−4.52 (−5.95, −3.06)^c^		1.55 (0.21, 2.90)^a^		−5.05 (−6.53, −3.55)^c^		1.86 (0.49, 3.26)^b^	
	Q3	−4.08 (−6.01, −2.10)^c^		1.18 (−0.27, 2.66)		−4.02 (−5.95, −2.04)^c^		1.72 (0.17, 3.30)^a^	
	Q4	−4.02 (−6.27, −1.72)^c^	0.008^b^	2.00 (0.14, 3.89)^a^	0.040^a^	−4.22 (−6.45, −1.94)^c^	0.008^b^	2.19 (0.32, 4.09)^a^	0.025^a^
	Per 10 µg/m^3^	−2.02 (−4.47, 0.49)		0.52 (−0.07, 1.12)		−2.19 (−4.63, 0.31)		0.56 (−0.04, 1.15)	
TBIL	Q1	Ref		Ref		Ref		Ref	
	Q2	1.76 (0.11, 3.43)^a^		3.34 (1.88, 4.83)^c^		0.76 (−0.93, 2.48)		2.78 (1.27, 4.30)^c^	
	Q3	−2.97 (−5.09, −0.80)^b^		4.21 (2.63, 5.82)^c^		−2.86 (−4.98, −0.69)^a^		3.22 (1.52, 4.96)^c^	
	Q4	1.37 (−1.20, 3.99)	0.008^b^	5.73 (3.68, 7.82)^c^	<0.001^c^	0.91 (−1.62, 3.52)	0.063	4.67 (2.22, 7.17)^c^	<0.001^c^
	Per 10 µg/m^3^	6.69 (3.75, 9.72)^c^		2.03 (1.25, 2.81)^c^		4.79 (1.97, 7.70)^c^		1.76 (1.12, 2.40)^c^	
Third trimester									
ALT	Q1	Ref		Ref		Ref		Ref	
	Q2	6.08 (3.83, 8.38)^c^		−0.65 (−2.59, 1.32)		7.41 (5.08, 9.80)^c^		−1.15 (−3.12, 0.86)	
	Q3	9.03 (6.16, 11.99)^c^		−1.16 (−3.30, 1.02)		7.93 (5.06, 10.88)^c^		−2.19 (−4.47, 0.14)	
	Q4	6.99 (3.71, 10.39)^c^	0.001^b^	−5.94 (−9.16, −2.59)^c^	0.003^b^	7.13 (3.85, 10.53)^c^	<0.001^c^	−6.58 (−9.86, −3.18)^c^	<0.001^c^
	Per 10 µg/m^3^	3.89 (0.32, 7.59)^a^		−2.73 (−3.86, −1.58)^c^		5.71 (2.06, 9.50)^b^		−3.15 (−4.27, −2.02)^c^	
AST	Q1	Ref		Ref		Ref		Ref	
	Q2	−3.70 (−4.86, −2.52)^c^		0.03 (−1.07, 1.15)		−3.81 (−5.00, −2.60)^c^		0.23 (−0.90, 1.37)	
	Q3	−3.48 (−4.92, −2.02)^c^		0.02 (−1.21, 1.27)		−3.38 (−4.84, −1.91)^c^		0.42 (−0.91, 1.77)	
	Q4	−3.14 (−4.82, −1.43)^c^	0.004^b^	0.07 (−1.86, 2.03)	0.961	−3.18 (−4.86, −1.47)^c^	0.004^b^	0.40 (−1.57, 2.41)	0.638
	Per 10 µg/m^3^	−1.68 (−3.59, 0.27)		−0.05 (−0.71, 0.61)		−1.73 (−3.66, 0.24)		0.07 (−0.61, 0.74)	
TBIL	Q1	Ref		Ref		Ref		Ref	
	Q2	1.31 (−0.30, 2.95)		1.46 (−0.01, 2.95)		1.49 (−0.18, 3.19)		0.43 (−1.06, 1.94)	
	Q3	3.89 (1.83, 6.00)^c^		0.07 (−1.52, 1.69)		3.78 (1.71, 5.90)^c^		−2.00 (−3.69, −0.28)^a^	
	Q4	8.57 (6.08, 11.12)^c^	<0.001^c^	−1.32 (−3.30, 0.70)	0.195	8.50 (6.01, 11.06)^c^	<0.001^c^	−2.76 (−5.20, −0.26)^a^	0.020^a^
	Per 10 µg/m^3^	9.27 (6.52, 12.10)^c^		−0.81 (−1.45, −0.17)^a^		9.72 (6.88, 12.64)^c^		−1.45 (−2.31, −0.59)^b^	

NO_2_ and O_3_ air pollution exposure during the first trimester.

Q1-Q4: First quartile, Second quartile, Third quartile, and Fourth quartile respectively.

Per 10 µg/m^3^: Analyzed as a continuous variable.

Model adjusted for mean temperature and relative humidity using natural cubic splines (*df* = 3), maternal age, gestational age at liver function testing, pregnancy methods, pre-pregnancy BMI, education, cigarette smoking, alcohol drinking, physical burden, gestational hypertension, gestational diabetes, status of HBsAg, taking medication during the first trimester, season at liver function testing.

^a^*P* < 0.05, ^b^*P* < 0.01, ^c^*P* < 0.001.

When both NO_2_ and O_3_ were categorized into quartiles, the findings from the two-pollutant model revealed a positive dose-response relationship between first-trimester NO_2_ exposure and second-trimester ALT level, as well as with third-trimester ALT and TBIL levels. Regarding first-trimester O_3_ exposure, a positive dose-response trend was observed with ALT levels during the second trimester, whereas a negative dose-response trend was noted with ALT and TBIL levels during the third trimester (Table 3). Furthermore, the nonlinear curve demonstrated a similar dose-response trend (Fig. S2 and Fig. S3).

### 3.3. Associations of air pollutant mixture with maternal liver function

**Fig. 1:**
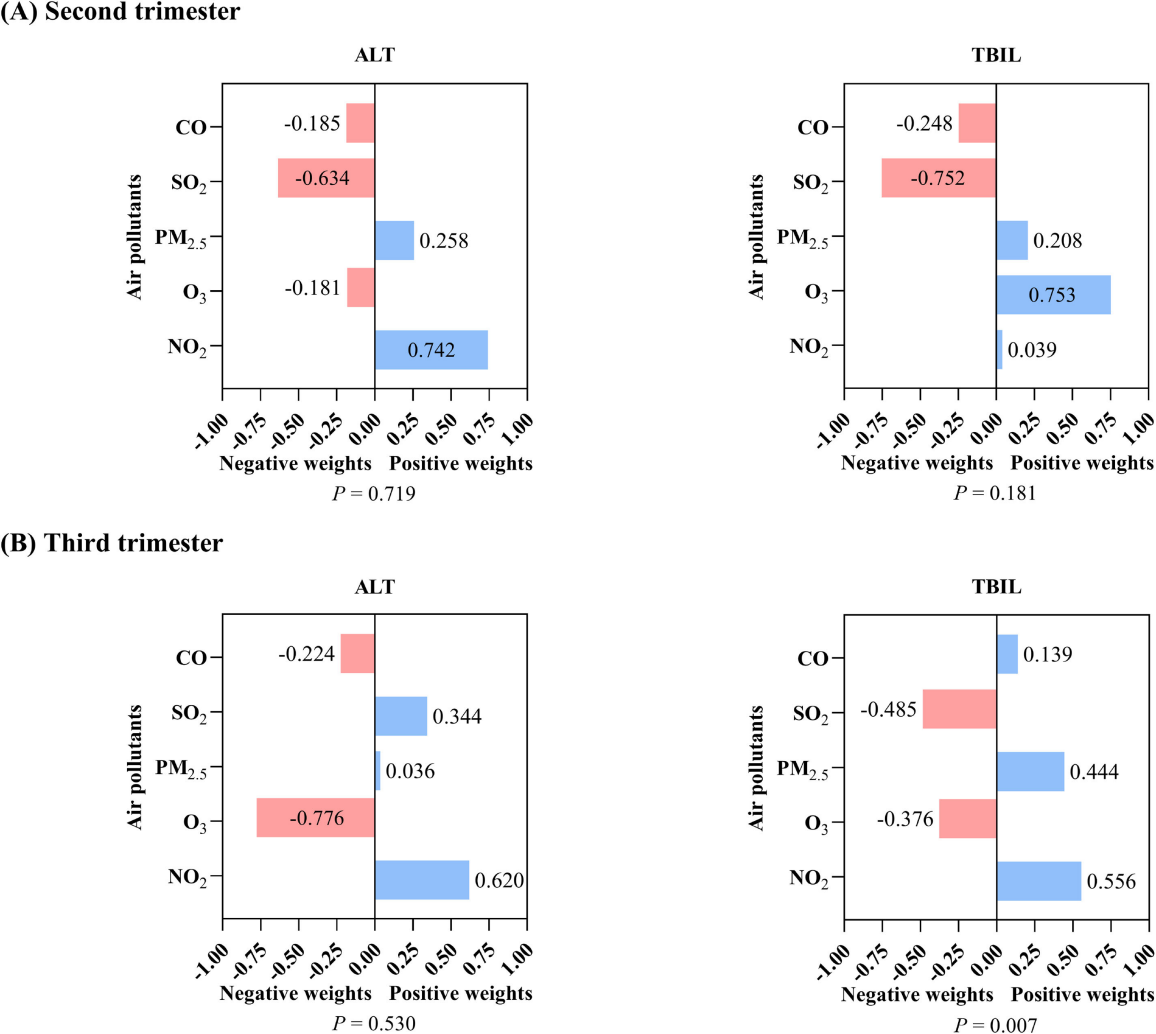
The weights of air pollutants in the mixed-exposure model (Qgcomp) Exposure to air pollutant mixture during the first trimester. Model adjusted for mean temperature, relative humidity, maternal age, gestational age at liver function testing, pregnancy methods, pre-pregnancy BMI, education, cigarette smoking, alcohol drinking, physical burden, gestational hypertension, gestational diabetes, status of HBsAg, taking medication during the first trimester, season at liver function testing.

Based on the results of the single-pollutant model and the two-pollutant model, we further used the quantile G-computation (Qgcomp) model to analyze the association of air pollutant mixture with ALT and TBIL levels. The air pollutant mixture included five major pollutants: NO_2_, O_3_, PM_2.5_, SO_2_ and CO, the correlation results of air pollutants is shown in Fig. S4. As presented in Table 4 and Fig. 1, Each one-quartile increase in first-trimester air pollutant mixture exposure was associated with a 2.05% (95% CI: 0.56%, 3.56%) increase in TBIL levels in the third trimester. In this model, NO_2_ accounted for 55.6% of the positive weight, while O_3_ accounted for 37.6% of the negative weight.

**Table 4:** The associations of exposure to air pollutant mixture with maternal liver function in Qgcomp model

Liver function biomarkers	Pollutant	*β* ^a^	Effect of mixture *ψ*^b^ (95%CI)	%Change (95%CI)	*P*
Second trimester					
ALT			0.005 (−0.023, 0.033)	0.51 (−2.23, 3.33)	0.719
	NO_2_	0.046			
	O_3_	−0.010			
	PM_2.5_	0.016			
	SO_2_	−0.036			
	CO	−0.010			

TBIL			−0.011 (−0.027, 0.005)	−1.09 (−2.67, 0.51)	0.181
	NO_2_	0.001			
	O_3_	0.020			
	PM_2.5_	0.005			
	SO_2_	−0.028			
	CO	−0.009			

Third trimester					
ALT			−0.008 (−0.031, 0.016)	−0.75 (−3.06, 1.61)	0.530
	NO_2_	0.028			
	O_3_	−0.040			
	PM_2.5_	0.002			
	SO_2_	0.015			
	CO	−0.012			

TBIL			0.020 (0.006, 0.035)	2.05 (0.56, 3.56)	0.007^c^
	NO_2_	0.019			
	O_3_	−0.005			
	PM_2.5_	0.015			
	SO_2_	−0.007			
	CO	−0.002			

Exposure to air pollutant mixture during the first trimester.

Qgcomp model: quantile G-computation model.

^a^*β* and ^b^*ψ* refers to the estimate obtained after logarithmic transformation of liver function biomarkers.

The %Change (95%CI) is calculated for per one-quartile increase in pollutant exposure.

Model adjusted for mean temperature, relative humidity, maternal age, gestational age at liver function testing, pregnancy methods, pre-pregnancy BMI, education, cigarette smoking, alcohol drinking, physical burden, gestational hypertension, gestational diabetes, status of HBsAg, season at liver function testing.

^c^*P* < 0.01

### 3.4. Associations of NO_2_ and O_3_ air pollution exposure with abnormal elevation of liver enzymes

Table S4 shows the association of NO_2_ and O_3_ with abnormal elevation of liver enzymes. After adjusting for variables, no association was found between NO_2_ or O_3_ and the increased risk of elevated maternal liver enzymes in either the single-pollutant model or the two-pollutant model.

### 3.5. Stratified analysis

**Fig. 2:**
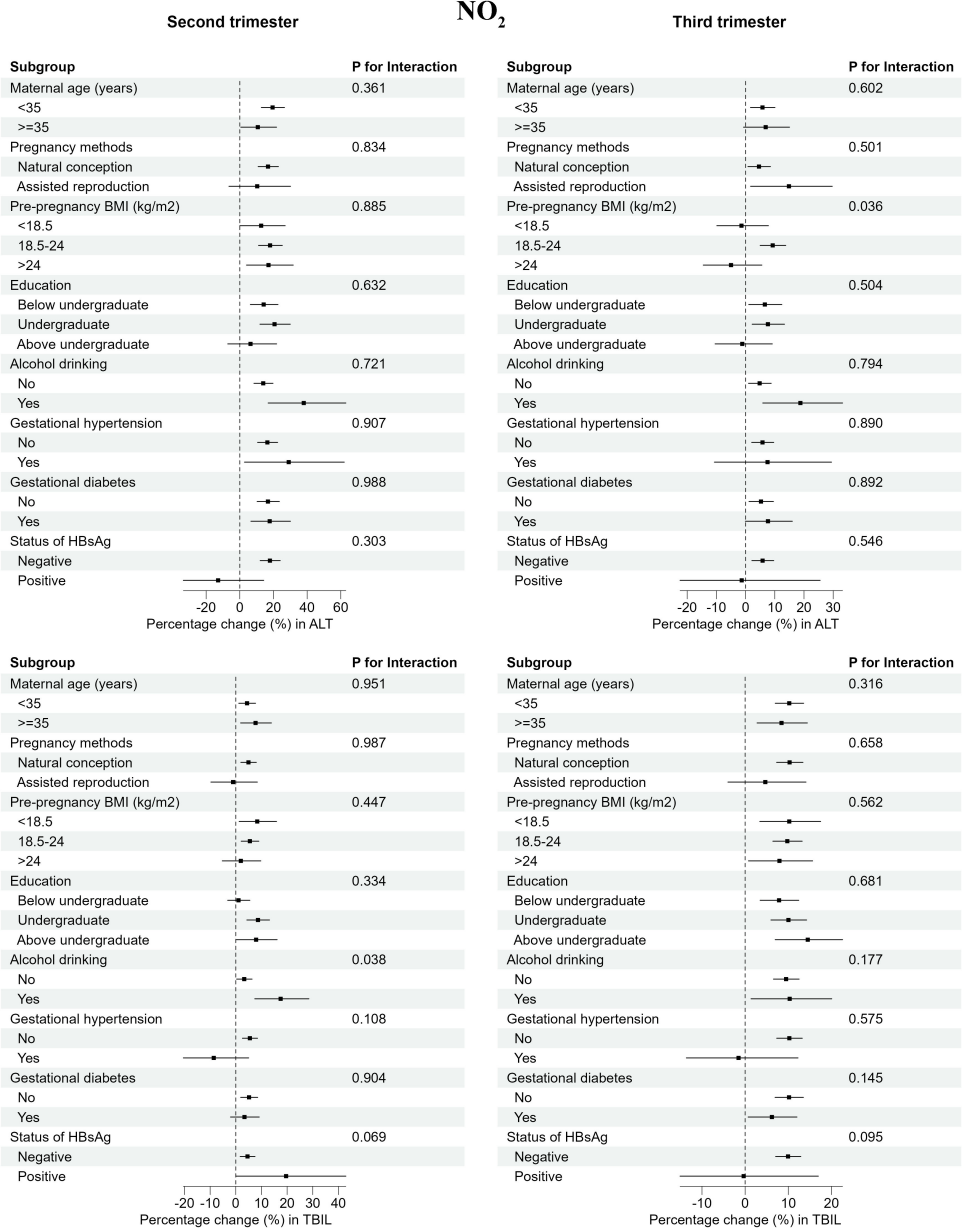
Stratified analysis of the association of NO_2_ air pollution exposure with maternal liver function NO_2_ air pollution exposure during the first trimester. The stratification factors include maternal age, pregnancy methods, pre-pregnancy BMI, education level, alcohol-drinking status, gestational hypertension, gestational diabetes, and status of HBsAg. The symbols show the percentage change (%) and the horizontal bars show the 95% confidence intervals.

**Fig. 3:**
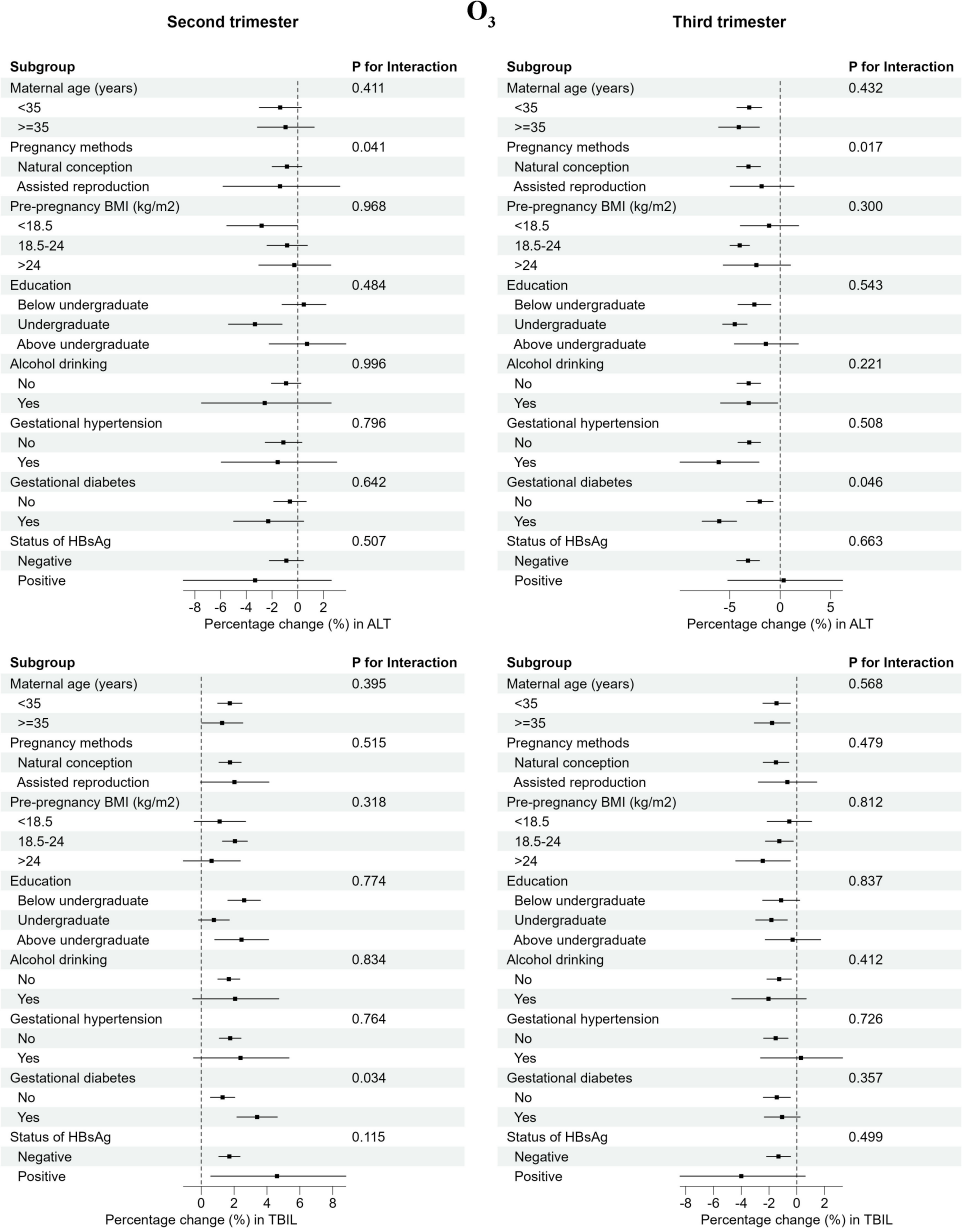
Stratified analysis of the association of O_3_ air pollution exposure with maternal liver function O_3_ air pollution exposure during the first trimester. The stratification factors include maternal age, pregnancy methods, pre-pregnancy BMI, education level, alcohol-drinking status, gestational hypertension, gestational diabetes, and status of HBsAg. The symbols show the percentage change (%) and the horizontal bars show the 95% confidence intervals.

Stratified analysis revealed a stronger positive association between NO_2_ exposure and second-trimester TBIL levels among pregnant women who drank alcohol. For pregnant women with normal BMI levels, NO_2_ exposure was associated with an increase in third-trimester ALT levels. Additionally, among pregnant women with natural conception, exposure to O_3_ was associated with a reduction in ALT levels, whereas no significant association was found between O_3_ and third-trimester ALT levels among pregnant women with assisted reproduction (*P* for Interaction <0.05) (Fig. 2 and Fig. 3).

### 3.6. Sensitivity analysis

We adjusted the *df* from 3 to 7 and found that, whether in the single-pollutant model or the two-pollutant model, our results did not show significant changes (Fig. S5 and Fig. S6). After excluding pregnant women with gestational diabetes or gestational hypertension separately, or either of these conditions, the results of our analysis still showed no significantly change (Fig. S7). We performed an analysis of the association between second-trimester air pollutant exposure and third-trimester maternal liver function to further verify the relatively short-term impacts of NO_2_ and O_3_ exposure on maternal liver function. The findings were similar to the association observed between first-trimester air pollutant exposure and second-trimester maternal liver function, which further confirms that the relatively short-term impacts of air pollutants on maternal liver function exhibit cross-trimester stability and consistency (Table S5). After adjusting for the cumulative exposure effects during the second and third trimesters using the two-period model, the results showed that the independent effect of first-trimester exposure remained stable (Table S6 and Table S7).

## 4. Discussion

Air pollutant exposure is significantly associated with adverse maternal health outcomes during pregnancy. In this retrospective birth cohort study, we investigated the associations between NO_2_ and O_3_ air pollution exposure in early pregnancy and liver function in pregnant women, and explored the contributions of NO_2_ and O_3_ in the combined effects of air pollutant mixture on maternal liver function. To our knowledge, this represents the first investigation to explore the associations between air pollutants and liver function in pregnant women by considering the combined effects of air pollutant mixture. The results demonstrated that first-trimester NO_2_ exposure was significantly and robustly associated with elevated levels of ALT and TBIL in the second and third trimesters. Regarding O_3_, first-trimester O_3_ exposure was related to increased TBIL levels in the second trimester, but associated with decreased ALT and TBIL levels in the third trimester. The air pollutant mixture model indicated that first-trimester exposure to the pollutant mixture was associated with increased TBIL during the third trimester, and NO_2_ may have a strong relative contribution to the combined association in the model. Additionally, alcohol drinking status may modify the associations of air pollutants with maternal liver function.

In this study, the average concentrations of NO_2_ and O_3_ to which pregnant women were exposed during the first trimester were 21.08 ± 3.54 µg/m^3^ and 93.40 ± 11.98 µg/m^3^, respectively. Globally, long-term NO_2_ monitoring data from 2000 to 2019 showed the following descending order of concentrations: China (16.9 ± 9.0 µg/m^3^) > India (15.5 ± 5.6 µg/m^3^) > United States (10.7 ± 5.6 µg/m^3^) > Europe (7.7 ± 4.5 µg/m^3^). Although the NO_2_ concentration in China decreased slightly at an annual rate of −0.23 µg/m^3^ from 2013 to 2019, it remained generally stable [18]. With respect to O_3_ pollution, global monitoring data from 2013 to 2022 identified the North China Plain (approximately 136.3 ± 9.2 µg/m^3^) and the Yangtze River Delta (120.5 ± 11.1 µg/m^3^) in China, as well as Southern California in the southwestern United States (107.9 ± 20.5 µg/m^3^), as core global hotspots of O_3_ pollution. In recent years, ambient O_3_ concentrations in China have shown an overall upward trend, with the growth rate slowing down after 2018 [17, 39]. Another global survey also noted that India, Mexico, China, South Korea, and Japan are the countries with the most frequent O_3_ standard exceedances [19]. Compared with the aforementioned global atmospheric pollutant levels, the NO_2_ concentration observed in this study was slightly higher than the national average in China. Although O_3_ concentrations were lower than those in O_3_ pollution hotspots in both China and the United States, it still shows an upward trend.

Multiple epidemiological studies have previously explored the relationships between air pollutant exposure and liver enzymes in both general and specific populations [21–23, 40–42]. For example, Li et al. conducted a longitudinal research with 318,911 elderly individuals to explore the association of air pollutants with serum liver enzymes. They discovered that exposure to ambient air pollutants such as PM_2.5_, NO_2_, and O_3_ was significantly associated with elevated levels of ALT (4.6%, 4.6%, 3.6%) and AST (4.6%, 3.3%, 4.0%) [21]. Another investigation focusing on individuals with HIV/AIDS revealed that short-term exposure to O_3_ was significantly correlated with elevated concentrations of ALT and AST [42]. Markevych et al. explored the associations between liver enzymes and air pollutants, such as particulate matter, NO_2_, and nitrogen oxides (NO_x_), within the 31–85-year-old adult group in the Augsburg region of Germany. They found that only PM_2.5_ was negatively associated with liver enzymes [40]. Additionally, a cohort study conducted in Foshan City, China, showed that exposure to PM_2.5_, O_3_, and SO_2_ during the second trimester was associated with increased ALT levels in the third trimester, whereas no such association was observed for NO_2_ exposure [23]. Although the findings of these studies are not entirely consistent, they collectively suggest associations between air pollutant exposure and changes in liver enzymes. Our study, which focused on pregnant women, found that first-trimester NO_2_ exposure was associated with increased ALT levels in the second and third trimesters, providing supplementary evidence supporting these associations. However, considering the differences in study populations, exposure concentrations, exposure durations, and potential variations in biological mechanisms, the results of these studies may not be directly comparable. Additional studies are needed to examine the associations between air pollutant exposure and liver enzymes.

Another critical biomarker of liver function is TBIL. Currently, there is still a lack of substantial evidence linking air pollutant to bilirubin, particularly TBIL. A study focusing on neonatal populations reported that PM_2.5_, CO and SO_2_ air pollution exposure was associated with elevated bilirubin levels in newborns [43]. Another research conducted in Taiwan indicated that maternal air pollutant exposure including NO_2_, CO and PM_2.5_, was associated with increased serum TBIL levels in neonates [44]. Our previous study also demonstrated a significant association between PM_2.5_ exposure and increased maternal TBIL during pregnancy [45]. In this study, we found that first-trimester NO_2_ exposure was associated with elevated TBIL levels in the second and third trimesters, while O_3_ exposure was associated with second-trimester TBIL elevation. Our findings are generally consistent with previous evidence on the association between air pollutant and TBIL. In addition, results from the air pollutant mixture model indicated that exposure to air pollutant mixture was associated with third-trimester TBIL elevation, with NO_2_ contributing 55.6% of the positive weight in the model. This substantial relative contribution suggests that NO_2_ may be an important component driving the model-based association between combined air pollutant exposure and increased TBIL levels in the third trimester. These findings support the need for greater public health attention to air pollution exposure during pregnancy.

In this study, no significant association was found between NO_2_ and O_3_ exposure and clinically abnormal elevation of liver enzyme levels, indicating that the observed biomarker changes may primarily remain at a subclinical level under the current exposure concentrations. However, within the relatively narrow exposure range of our study, clear dose–response relationships were still observed between NO_2_ and O_3_ exposure and continuous liver function biomarkers, suggesting that further increases in exposure concentrations may be associated with clinical abnormalities in maternal liver function. This inference is supported by recent research evidence. A previous study reported an NO_2_ concentration of 33.6 ± 5.3 µg/m^3^, which was substantially higher than the exposure levels in our study, and confirmed significant associations of NO_2_ with both clinically abnormal liver function and continuous biomarker alterations under such elevated exposure conditions [21]. This supports the potential clinical relevance of the subclinical changes observed in our study under high-exposure scenarios. From a clinical perspective, dynamic monitoring of fluctuations in liver function biomarkers in the context of air pollution exposure may help identify early signals to guide timely intervention, especially in regions with high pollution levels. Nevertheless, future studies covering a broader range of air pollution exposure levels are needed to further validate this viewpoint.

The toxic mechanisms of air pollutants on the liver remain unclear. Current research suggests that oxidative stress is widely recognized as a major contributor through which air pollutants induce liver toxicity [46, 47]. Under conditions of normal physiology, the oxidative/anti-oxidative stress system within the organism maintains a dynamic equilibrium state. Upon exposure to air pollutants, cells can be induced to generate a large quantity of reactive oxygen species (ROS), leading to an imbalance in the cellular oxidative/anti-oxidative system and subsequently triggering oxidative stress [48, 49]. During the process of oxidative stress, ROS can also promote the expression of inflammatory factors, triggering inflammatory responses, which further exacerbates the damage to liver cells and thus disrupts the integrity of the liver cell membrane [50]. Under such damaging effects, liver function is ultimately elevated. Moreover, as an endogenous antioxidant [51, 52], TBIL may increase its expression in order to maintain the oxidative/anti-oxidative balance under the stress of air pollutants. It participates synergistically in the antioxidant response mediated by cells, thereby alleviating the toxic impacts of air pollutants on the liver. These hypotheses provide a plausible explanation for our findings. However, the specific biological mechanisms still require further in-depth exploration.

In our investigation on the relationship between O_3_ and maternal liver function, we observed inconsistent associations across trimesters and biomarkers, specifically positive associations with second-trimester TBIL but inverse associations with third-trimester ALT and TBIL. The inverse findings are consistent with two previous studies [53, 54]. A study conducted in Hong Kong and Taiwan reported a negative correlation between O_3_ and the prevalence of non-alcoholic fatty liver disease (NAFLD) and advanced fibrosis [54]. In our research, O_3_ and NO_2_ were found to be negatively correlated, consistent with the findings of the aforementioned research, although the negative correlation in our study was relatively weak (Pearson’s r = −0.069, *P* < 0.05). Another study carried out in Wuhan and Zhuhai reported a negative association between O_3_ and liver enzymes [53]. Overall, according to the World Health Organization (WHO) Global Air Quality Guidelines and the Chinese Ambient Air Quality Standards [55–57], the O_3_ concentrations in these three studies were all at relatively low levels (Daily 8-hour average concentration <100 µg/m^3^). Previous experimental toxicological studies have suggested that low-concentration O_3_ may trigger adaptive cellular responses by activating the Nrf2 antioxidant stress signaling pathway and inhibiting inflammatory pathways, thereby alleviating liver injury [58]. Therefore, adaptive antioxidant responses may be a potential mechanism for the inverse associations between O_3_ and third-trimester ALT and TBIL in our results. However, we explicitly emphasize that this remains a speculative hypothesis and requires confirmation by further research evidence. The other alternative explanations for the observed inverse associations should also be considered, including potential residual confounding (e.g., unmeasured maternal diet, lifestyle, or occupational factors), seasonal variations in both ambient O_3_ levels and baseline liver function, limited exposure contrast within our cohort, and correlations with co-pollutants that may partially drive the observed patterns. Additionally, we further hypothesize that the liver defensive mechanisms in pregnant women against O_3_ exposure may exhibit time-dependent characteristics. Our sensitivity analysis showed that, within the same time span, the association between second-trimester O_3_ exposure and third-trimester liver function was similar to that between first-trimester O_3_ exposure and second-trimester liver function. However, when the exposure time span was extended, the association between O_3_ exposure and liver function throughout pregnancy underwent significant changes. Currently, direct evidence supporting these hypotheses is lacking. Future longitudinal and experimental studies are needed to elucidate the time-dependent effects of O_3_ exposure and to clarify the potential biological mechanisms underlying the differential associations with O_3_ exposure across different trimesters of pregnancy.

In this study, we observed a stronger relationship between NO_2_ exposure and elevated liver function during the second trimester among pregnant women who drank alcohol, suggesting that alcohol-drinking individuals are more vulnerable to the NO_2_ air pollution exposure. Similar results were presented in a study conducted in Southwest China in 2021 and a study carried out in Shenzhen in 2022 [21, 59]. Earlier research has indicated that alcohol can cause damage to liver cells and might interact with air pollutant exposure, leading to deviations in the levels of liver function biomarkers [60]. Additionally, we did not observe significant differences in the strength of the association between air pollutants and elevated liver function among pregnant women with natural conception and those with assisted reproductive technology. However, in pregnant women with natural conception, the liver function levels during the third trimester decreased with O_3_ exposure, potentially due to a heightened sensitivity of their protective mechanisms. Notably, the relatively high prevalence of gestational diabetes in our cohort reflects the nature of the study setting. The hospital where this research was conducted is a nationally renowned tertiary grade A hospital and a leading institution in regional maternal and child healthcare, which routinely admits high-risk pregnant women and patients with complicated or severe pregnancy-related conditions referred from lower-level medical institutions. Correspondingly, we also performed stratified analysis based on the status of gestational diabetes, and the results showed that gestational diabetes did not significantly modify the associations between NO_2_ and O_3_ exposure and maternal liver function.

Our study has several strengths, including a birth cohort design, large sample size, and consideration of more potential confounding factors. In addition, this is the first study to assess the associations between air pollutant and liver function in pregnant women by considering the combined effects of air pollutant mixtures. However, several limitations should be acknowledged. First, although this study was conducted in Shenzhen, a major immigrant city in China where the pregnant women in the study were from all parts of the country, it remains a single-center study with a relatively narrow range of air pollution exposure levels, which may limit the external validity of the findings. Second, air pollution exposure was assessed solely according to residential addresses, without accounting for factors including residential mobility, commuting, occupational exposure, time-activity patterns and indoor pollution, which may result in exposure misclassification bias. This bias is particularly concerning during pregnancy, as pregnant women frequently experience residential relocation, changes in commuting habits, reduced outdoor activity time, and alterations in occupational exposure throughout gestation. These dynamic factors may lead to systematic differences between exposure estimates based solely on residential addresses and true exposure levels, thereby potentially introducing unpredictable bias into the effect estimates. Third, the measurement of liver function may be subject to variability, and it is impossible to avoid the changes caused by other pathological or physiological factors. Finally, although our study has adjusted for some confounding factors, potential residual confounding cannot be entirely ruled out. Liver function biomarkers are sensitive not only to dietary intake, infection status, medication use, and pregnancy-related conditions, but also to factors such as nutritional status, occupational exposures, passive smoking, and physical activity. Notably, although we adjusted for physical burden and medication use, the classification of physical burden was relatively crude, and detailed information on medication type, dosage, and duration of use was lacking. This incomplete adjustment may introduce residual confounding, thereby affecting the accuracy of the effect estimates.

## 5. Conclusions

In conclusion, this study found that first-trimester NO_2_ exposure was significantly associated with elevated levels of ALT and TBIL in the second and third trimesters. First-trimester O_3_ exposure was associated with increased TBIL levels in the second trimester but associated with reduced ALT and TBIL levels in the third trimester, suggesting that the direction of the associations with O_3_ exposure may vary across different trimesters of pregnancy. In the mixed-pollutant model, the air pollutant mixture was associated with elevated TBIL levels in the third trimester, and NO_2_ may be the primary relative contributing component in the model. These findings indicate that NO_2_ exposure may play an important role in this model-based association between air pollutant exposure and liver function in pregnant women. Future research should include multicenter cohort studies with more refined exposure assessment methods and a wider range of air pollution exposure levels. Further efforts are needed to investigate the time-dependent effects of air pollutant exposure during pregnancy, and to clarify the biological mechanisms underlying the differential associations of O_3_ exposure across gestational stages. Our findings support the need for further studies and public health attention to air pollution exposure during pregnancy, which may help inform strategies to maintain stable liver function and protect maternal and fetal health.

## Supplementary information

**Additional file 1:** **Table S1.** General characteristics of included and excluded pregnant women (N = 25,208). **Table S2.** Questionnaire. **Table S3.** The linear associations of NO_2_ and O_3_ air pollution exposure with maternal liver function in the crude model. **Table S4.** The associations of NO_2_ and O_3_ air pollution exposure (per 10 µg/m^3^ increase) in the first trimester with abnormal elevation of liver enzymes (ALT or AST > 40 (U/L)) in the second and third trimesters. **Table S5.** The associations of second-trimester NO_2_ and O_3_ air pollution exposure with third-trimester maternal liver function in the two-pollutant model. **Table S6.** Sensitivity analysis of the association between first-trimester NO_2_ and O_3_ air pollution exposure and second-trimester maternal liver function in the two-period model. **Table S7.** Sensitivity analysis of the association between first-trimester NO_2_ and O_3_ air pollution exposure and third-trimester maternal liver function in the two-period model. **Fig. S1.** Inclusion and exclusion criteria for participants. **Fig. S2.** Non-linear associations between NO_2_ air pollution exposure in the first trimester and maternal liver function in the second and third trimesters. **Fig. S3.** Non-linear associations between O_3_ air pollution exposure in the first trimester and maternal liver function in the second and third trimesters. **Fig. S4.** Correlation of air pollutants. **Fig. S5.** Sensitivity analyses of associations of NO_2_ and O_3_ air pollution exposure in the first trimester with maternal liver function in the second and third trimesters by changing the degrees of freedom for the spline function from 4 to 7 in the single-pollutant model. **Fig. S6.** Sensitivity analyses of associations of NO_2_ and O_3_ air pollution exposure in the first trimester with maternal liver function in the second and third trimesters by changing the degrees of freedom for the spline function from 4 to 7 in the two-pollutant model. **Fig. S7.** Sensitivity analyses of associations of NO_2_ and O_3_ air pollution exposure in the first trimester with maternal liver function in the second and third trimesters by statuses of gestational hypertension and gestational diabetes.

## Declarations

### Availability of data and materials

Data will be made available on reasonable request. All inquiries about data should be sent to fanfan20221125@163.com.
