# Supplementary material for: Combined effects of nitrogen dioxide and ozone air pollution on maternal liver function during pregnancy: a birth cohort study in China
Source: Environ Health Prev Med. 2026 Jun 12;31:38. doi: 10.1265/ehpm.26-00057 (PMC13290539; doi:10.1265/ehpm.26-00057)
Supplement: Supplementary file 1 — Additional file 1: Table S1. General characteristics of included and excluded pregnant women (N = 25,208). Table S2. Questionnaire. Table S3. The linear associations of NO2 and O3 air pollution exposure with maternal liver function in the crude model. Table S4. The associations of NO2 and O3 air pollution exposure (per 10 µg/m3 increase) in the first trimester with abnormal elevation of liver enzymes (ALT or AST > 40 (U/L)) in the second and third trimesters. Table S5. The associations of second-trimester NO2 and O3 air pollution exposure with third-trimester maternal liver function in the two-pollutant model. Table S6. Sensitivity analysis of the association between first-trimester NO2 and O3 air pollution exposure and second-trimester maternal liver function in the two-period model. Table S7. Sensitivity analysis of the association between first-trimester NO2 and O3 air pollution exposure and third-trimester maternal liver function in the two-period model. Fig. S1. Inclusion and exclusion criteria for participants. Fig. S2. Non-linear associations between NO2 air pollution exposure in the first trimester and maternal liver function in the second and third trimesters. Fig. S3. Non-linear associations between O3 air pollution exposure in the first trimester and maternal liver function in the second and third trimesters. Fig. S4. Correlation of air pollutants. Fig. S5. Sensitivity analyses of associations of NO2 and O3 air pollution exposure in the first trimester with maternal liver function in the second and third trimesters by changing the degrees of freedom for the spline function from 4 to 7 in the single-pollutant model. Fig. S6. Sensitivity analyses of associations of NO2 and O3 air pollution exposure in the first trimester with maternal liver function in the second and third trimesters by changing the degrees of freedom for the spline function from 4 to 7 in the two-pollutant model. Fig. S7. Sensitivity analyses of associations of NO2 and O3 air pollution exp [file ehpm-31-038-s001.docx]

Supplementary Material

Combined effects of nitrogen dioxide and ozone air pollution on maternal liver function during pregnancy: a birth cohort study in China

Zhijian Chen ^a, b, 1^, Zhongai Ouyang ^c^, Weigui Ni ^b,^, Bingyi Lin ^b^, Haoqu Zheng ^a^, Long Jiang ^b^, Bo Wu ^d^, Lijuan Lai ^b^, Minting Zhu ^b^, Yi Jing ^b^, Zhuang Liu ^a^, Xi Yu ^a,^ *, Jingjie Fan ^b, c,^ *

^a^ Faculty of Medicine, Macau University of Science and Technology, Avenida Wai Long, Taipa, Macau, China

^b^ Department of Preventive Healthcare, Shenzhen Maternity and Child Healthcare Hospital, Women and Children's Medical Center, Southern Medical University, Shenzhen 518028, China

^c^ School of Public Health, Southern Medical University, Guangzhou 510515, China

^d^ Department of Dermatology, Shenzhen Maternity and Child Healthcare Hospital, Women and Children's Medical Center, Southern Medical University, Shenzhen 518028, China

Supplementary text

Supplementary tables

Table S1. General characteristics of included and excluded pregnant women (N = 25,208)

Table S2. Questionnaire

Table S3. The linear associations of NO_2_ and O_3_ air pollution exposure with maternal liver function in the crude model

Table S4. The associations of NO_2_ and O_3_ air pollution exposure (per 10 µg/m^3^ increase) in the first trimester with abnormal elevation of liver enzymes (ALT or AST > 40 (U/L)) in the second and third trimesters

Table S5. The associations of second-trimester NO_2_ and O_3_ air pollution exposure with third-trimester maternal liver function in the two-pollutant model

Table S6. Sensitivity analysis of the association between first-trimester NO_2_ and O_3_ air pollution exposure and second-trimester maternal liver function in the two-period model

Table S7. Sensitivity analysis of the association between first-trimester NO_2_ and O_3_ air pollution exposure and third-trimester maternal liver function in the two-period model

Supplementary figures

Fig. S1. Inclusion and exclusion criteria for participants

Fig. S2. Non-linear associations between NO_2_ air pollution exposure in the first trimester and maternal liver function in the second and third trimesters

Fig. S3. Non-linear associations between O_3_ air pollution exposure in the first trimester and maternal liver function in the second and third trimesters

Fig. S4. Correlation of air pollutants

Fig. S5. Sensitivity analyses of associations of NO_2_ and O_3_ air pollution exposure in the first trimester with maternal liver function in the second and third trimesters by changing the degrees of freedom for the spline function from 4 to 7 in the single-pollutant model

Fig. S6. Sensitivity analyses of associations of NO_2_ and O_3_ air pollution exposure in the first trimester with maternal liver function in the second and third trimesters by changing the degrees of freedom for the spline function from 4 to 7 in the two-pollutant model

Fig. S7. Sensitivity analyses of associations of NO_2_ and O_3_ air pollution exposure in the first trimester with maternal liver function in the second and third trimesters by statuses of gestational hypertension and gestational diabetes

Table S1 General characteristics of included and excluded pregnant women (N = 25,208)

| Characteristics  Mean ± SD or N (%) | Included group  (N = 11,909) | Excluded group  (N = 13,299) | *P* |
| --- | --- | --- | --- |
| Maternal age (years) | 31.45 ± 4.06 | 31.28 ± 4.28 | 0.001^b^ |
| Pre-pregnancy BMI (kg/m^2^) | 21.02 ± 2.73 | 21.05 ± 2.81 | 0.322 |
| Pregnancy methods |  |  | 0.039^a^ |
| Natural conception | 10834 (90.97) | 11996 (90.20) |  |
| Assisted reproduction | 1075 (9.03) | 1303 (9.80) |  |
| Education |  |  | 0.236 |
| Below undergraduate | 4865 (40.85) | 5542 (41.68) |  |
| Undergraduate | 5413 (45.45) | 6018 (45.25) |  |
| Above undergraduate | 1631 (13.70) | 1739 (13.07) |  |
| Cigarette smoking |  |  | 0.483 |
| Never | 11666 (97.96) | 13003 (97.77) |  |
| Current | 36 (0.30) | 50 (0.38) |  |
| Former | 207 (1.74) | 246 (1.85) |  |
| Alcohol drinking |  |  | 0.242 |
| No | 10830 (90.94) | 12036 (90.50) |  |
| Yes | 1079 (9.06) | 1263 (9.50) |  |
| Physical burden |  |  | 0.095 |
| Light | 7952 (66.77) | 8764 (65.91) |  |
| Medium | 3909 (32.83) | 4459 (33.53) |  |
| Heavy | 48 (0.40) | 74 (0.56) |  |
| Gestational hypertension |  |  | 0.005^b^ |
| No | 11333 (95.16) | 12752 (95.89) |  |
| Yes | 576 (4.84) | 546 (4.11) |  |
| Gestational diabetes |  |  | 0.064 |
| No | 9150 (76.83) | 10349 (77.82) |  |
| Yes | 2759 (23.17) | 2950 (22.18) |  |
| Status of HBsAg |  |  | 0.374 |
| Negative | 11569 (97.15) | 12893 (96.95) |  |
| Positive | 340 (2.85) | 406 (3.05) |  |
| Taking medication during the first trimester |  |  | 0.986 |
| No | 7310 (61.38) | 8157 (61.34) |  |
| Yes | 4599 (38.62) | 5136 (38.66) |  |

Some participants in the excluded group did not undergo liver function testing. Therefore, no comparative analysis was performed between the two groups for the variables of gestational age at liver function testing (weeks) and season of liver function testing.

^a^ *P* < 0.05, ^b^ *P* < 0.01.

Table S2 Questionnaire

| Questions | Options |
| --- | --- |
| Basic information |  |
| Maternal age (years) | - |
| Height (cm) | - |
| Pre-pregnancy weight (kg) | - |
| Pregnancy methods | Natural conception/Assisted reproduction |
| Education | Below undergraduate/Undergraduate/Above undergraduate |
| Lifestyle |  |
| History of Smoking | Never/Current/Former |
| History of Alcohol drinking | No/Yes |
| Physical burden* | Light/Medium/Heavy |
| Taking medication during the first trimester | No/Yes |

*Physical burden: the overall amount of physical work an individual performs daily across different activities, such as work, household chores, sports, and recreational activities.

Table S3 The linear associations of NO_2_ and O_3_ air pollution exposure with maternal liver function in the crude model

|  |  | Crude model | | | | |
| --- | --- | --- | --- | --- | --- | --- |
|  |  | NO_2_ |  |  | O_3_ |  |
| Liver function biomarkers | Quartiles and continuous measure | %Change (95%CI) | *P* for trend |  | %Change (95%CI) | *P* for trend |
| Second trimester |  |  |  |  |  |  |
| ALT | Q1 | Ref |  |  | Ref |  |
|  | Q2 | 3.07 (0.29, 5.92)^a^ |  |  | 1.19 (-1.34, 3.79) |  |
|  | Q3 | 10.43 (6.74, 14.25)^c^ |  |  | 3.30 (0.46, 6.22)^a^ |  |
|  | Q4 | 12.91 (8.55, 17.44)^c^ | <0.001^c^ |  | 2.97 (-1.31, 7.42) | 0.059 |
|  | Per 10 µg/m^3^ | 17.10 (12.10, 22.32)^c^ |  |  | -1.10 (-2.19, 0.01) |  |
| AST | Q1 | Ref |  |  | Ref |  |
|  | Q2 | -4.62 (-6.03, -3.20)^c^ |  |  | 1.57 (0.19, 2.97)^a^ |  |
|  | Q3 | -4.18 (-5.93, -2.39)^c^ |  |  | 0.82 (-0.66, 2.33) |  |
|  | Q4 | -3.90 (-5.93, -1.82)^c^ | 0.003^b^ |  | 1.68 (-0.23, 3.61) | 0.104 |
|  | Per 10 µg/m^3^ | -2.29 (-4.58, 0.05) |  |  | 0.39 (-0.21, 1.00) |  |
| TBIL | Q1 | Ref |  |  | Ref |  |
|  | Q2 | 1.34 (-0.23, 2.94) |  |  | 3.42 (1.95, 4.92)^c^ |  |
|  | Q3 | -4.01 (-5.86, -2.11)^c^ |  |  | 3.94 (2.35, 5.55)^c^ |  |
|  | Q4 | 0.20 (-2.05, 2.49) | 0.219 |  | 5.52 (3.47, 7.61)^c^ | <0.001^c^ |
|  | Per 10 µg/m^3^ | 3.89 (1.28, 6.58)^b^ |  |  | 2.17 (1.33, 3.02)^c^ |  |
| Third trimester |  |  |  |  |  |  |
| ALT | Q1 | Ref |  |  | Ref |  |
|  | Q2 | 5.52 (3.31, 7.78)^c^ |  |  | -0.88 (-2.83, 1.11) |  |
|  | Q3 | 8.01 (5.18, 10.92)^c^ |  |  | -1.51 (-3.63, 0.65) |  |
|  | Q4 | 5.83 (2.60, 9.15)^c^ | 0.008^b^ |  | -6.07 (-9.29, -2.74)^c^ | 0.002^b^ |
|  | Per 10 µg/m^3^ | 3.06 (-0.49, 6.74) |  |  | -2.82 (-3.96, -1.66)^c^ |  |
| AST | Q1 | Ref |  |  | Ref |  |
|  | Q2 | -3.80 (-4.97, -2.61)^c^ |  |  | -0.02 (-1.15, 1.12) |  |
|  | Q3 | -3.58 (-5.03, -2.11)^c^ |  |  | -0.15 (-1.39, 1.10) |  |
|  | Q4 | -3.11 (-4.80, -1.38)^c^ | 0.006^b^ |  | -0.16 (-2.11, 1.82) | 0.823 |
|  | Per 10 µg/m^3^ | -1.77 (-3.70, 0.21) |  |  | -0.22 (-0.89, 0.45) |  |
| TBIL | Q1 | Ref |  |  | Ref |  |
|  | Q2 | 0.73 (-0.86, 2.34) |  |  | 1.40 (-0.08, 2.89) |  |
|  | Q3 | 3.31 (1.27, 5.39)^b^ |  |  | -0.36 (-1.93, 1.24) |  |
|  | Q4 | 7.89 (5.44, 10.40)^c^ | <0.001^c^ |  | -1.94 (-3.90, 0.05) | 0.049^a^ |
|  | Per 10 µg/m^3^ | 8.71 (5.97, 11.52)^c^ |  |  | -0.98 (-1.62, -0.35)^b^ |  |

Q1-Q4: First quartile, Second quartile, Third quartile, and Fourth quartile respectively.

NO_2_ and O_3_ air pollution exposure during the first trimester.

Crude model adjusted only for mean temperature and relative humidity using natural cubic splines (*df* = 3).

^a^ *P* < 0.05, ^b^ *P* < 0.01, ^c^ *P* < 0.001.

Table S4 The associations of NO_2_ and O_3_ air pollution exposure (each 10 µg/m^3^ increase) in the first trimester with abnormal elevation of liver enzymes (ALT or AST > 40 (U/L)) in the second and third trimesters

|  | Elevated liver enzymes (ALT or AST > 40 (U/L)) | |
| --- | --- | --- |
|  | Second trimester  OR (95%CI) | Third trimester  OR (95%CI) |
| Single-pollutant model |  |  |
| NO_2_ | 0.85 (0.53, 1.37) | 0.68 (0.30, 1.55) |
| O_3_ | 1.06 (0.95, 1.18) | 1.02 (0.92, 1.13) |
| Two-pollutant model |  |  |
| NO_2_ | 0.85 (0.53, 1.36) | 0.91 (0.40, 2.07) |
| O_3_ | 1.06 (0.96, 1.18) | 1.05 (0.95, 1.17) |

NO_2_ and O_3_ air pollution exposure during the first trimester.

Model adjusted for mean temperature and relative humidity using natural cubic splines (*df* = 3), maternal age, gestational age at liver function testing, pregnancy methods, pre-pregnancy BMI, education, cigarette smoking, alcohol drinking, physical burden, gestational hypertension, gestational diabetes, status of HBsAg, taking medication during the first trimester, season at liver function testing.

Table S5 The associations of second-trimester NO_2_ and O_3_ air pollution exposure with third-trimester maternal liver function in the two-pollutant model

| Liver function biomarkers | Two-pollutant model | | |
| --- | --- | --- | --- |
|  | NO_2_  %Change (95%CI) |  | O_3_  %Change (95%CI) |
| Third trimester |  |  |  |
| ALT | 19.60 (14.40, 25.03)^b^ |  | 0.89 (-0.36, 2.15) |
| AST | -2.04 (-4.36, 0.34) |  | 0.91 (0.21, 1.61)^a^ |
| TBIL | 4.26 (0.81, 7.82)^a^ |  | 0.91 (0.12, 1.71)^a^ |

NO_2_ and O_3_ air pollution exposure during the second trimester.

The %Change (95%CI) is calculated for each 10 μg/m^3^ increase in pollutant exposure.

Model adjusted for mean temperature and relative humidity using natural cubic splines (*df* = 3), maternal age, gestational age at liver function testing, pregnancy methods, pre-pregnancy BMI, education, cigarette smoking, alcohol drinking, physical burden, gestational hypertension, gestational diabetes, status of HBsAg, season at liver function testing.

^a^ *P* < 0.05, ^b^ *P* < 0.001.

Table S6 Sensitivity analysis of the association between first-trimester NO_2_ and O_3_ air pollution exposure and second-trimester maternal liver function in the two-period model

| Liver function biomarkers | NO_2_ |  | O_3_ |
| --- | --- | --- | --- |
|  | %Change (95%CI) |  | %Change (95%CI) |
| Second trimester |  |  |  |
| ALT | 10.07 (4.42, 16.02)^b^ |  | -0.65 (-1.74, 0.45) |
| AST | -0.20 (-2.97, 2.65) |  | 0.52 (-0.07, 1.12) |
| TBIL | 9.15 (5.89, 12.51)^b^ |  | 1.56 (0.79, 2.33)^b^ |

NO_2_ and O_3_ air pollution exposure during the first trimester.

The %Change (95%CI) is calculated for each 10 μg/m^3^ increase in pollutant exposure.

Model adjusted for mean temperature and relative humidity using natural cubic splines (*df* = 3), maternal age, gestational age at liver function testing, pregnancy methods, pre-pregnancy BMI, education, cigarette smoking, alcohol drinking, physical burden, gestational hypertension, gestational diabetes, status of HBsAg, season at liver function testing, second-trimester exposure concentrations of air pollutants (NO_2_/O_3_).

^a^ *P* < 0.05, ^b^ *P* < 0.001.

Table S7 Sensitivity analysis of the association between first-trimester NO_2_ and O_3_ air pollution exposure and third-trimester maternal liver function in the two-period model

| Liver function biomarkers | NO_2_ |  | O_3_ |
| --- | --- | --- | --- |
|  | %Change (95%CI) |  | %Change (95%CI) |
| Third trimester |  |  |  |
| ALT | 6.64 (2.48, 10.97)^b^ |  | -2.77 (-4.15, -1.36)^c^ |
| AST | -0.47 (-2.68, 1.79) |  | 0.37 (-0.65, 0.73) |
| TBIL | 10.15 (6.94, 13.46)^c^ |  | -1.69 (-2.74, -0.62)^b^ |

NO_2_ and O_3_ air pollution exposure during the first trimester.

The %Change (95%CI) is calculated for each 10 μg/m^3^ increase in pollutant exposure.

Model adjusted for mean temperature and relative humidity using natural cubic splines (*df* = 3), maternal age, gestational age at liver function testing, pregnancy methods, pre-pregnancy BMI, education, cigarette smoking, alcohol drinking, physical burden, gestational hypertension, gestational diabetes, status of HBsAg, season at liver function testing, average concentrations of air pollutants during the second and third trimesters (NO_2_/O_3_).

^a^ *P* < 0.05, ^b^ *P* < 0.01, ^c^ *P* < 0.001.


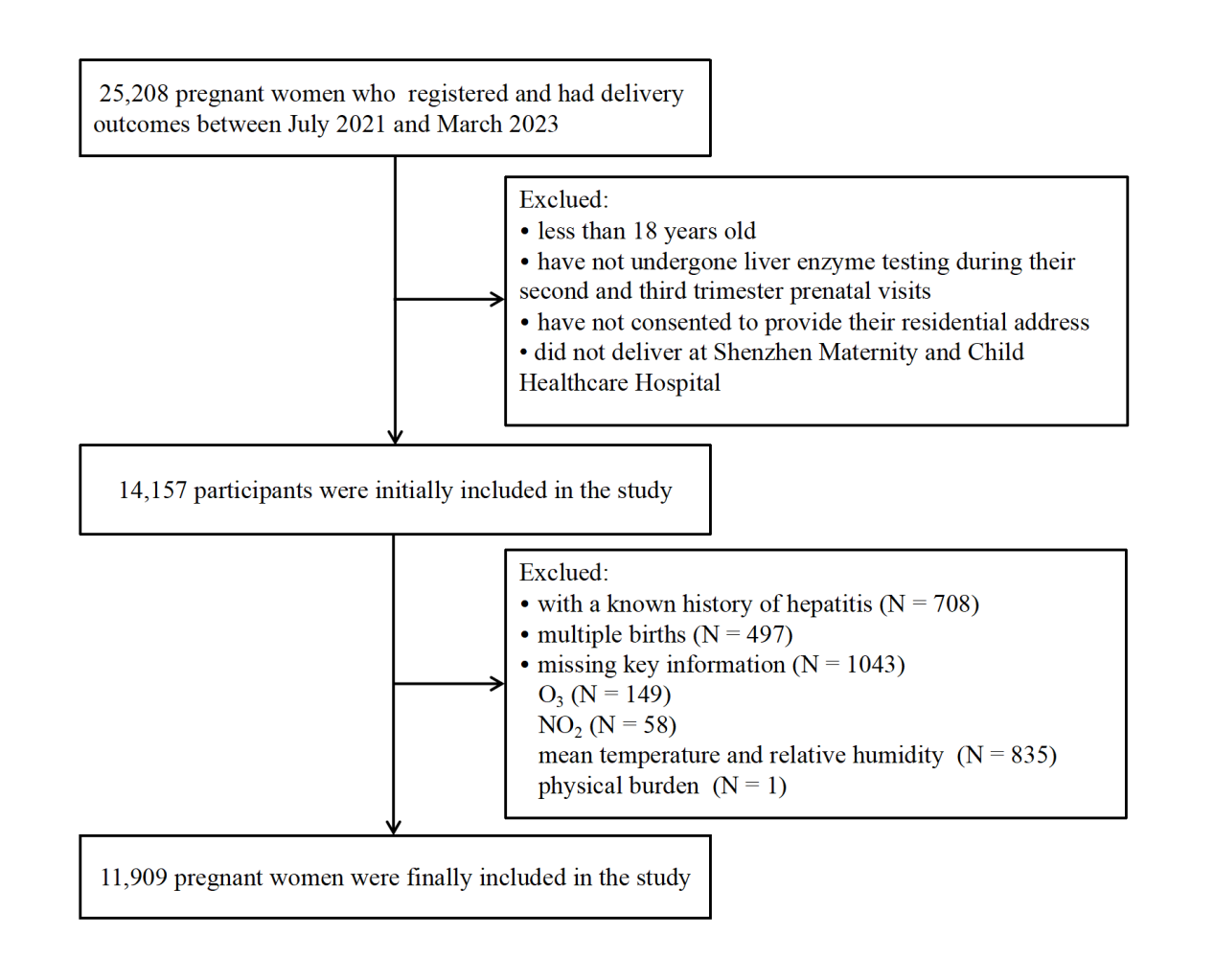
Fig. S1 Inclusion and exclusion criteria for participants


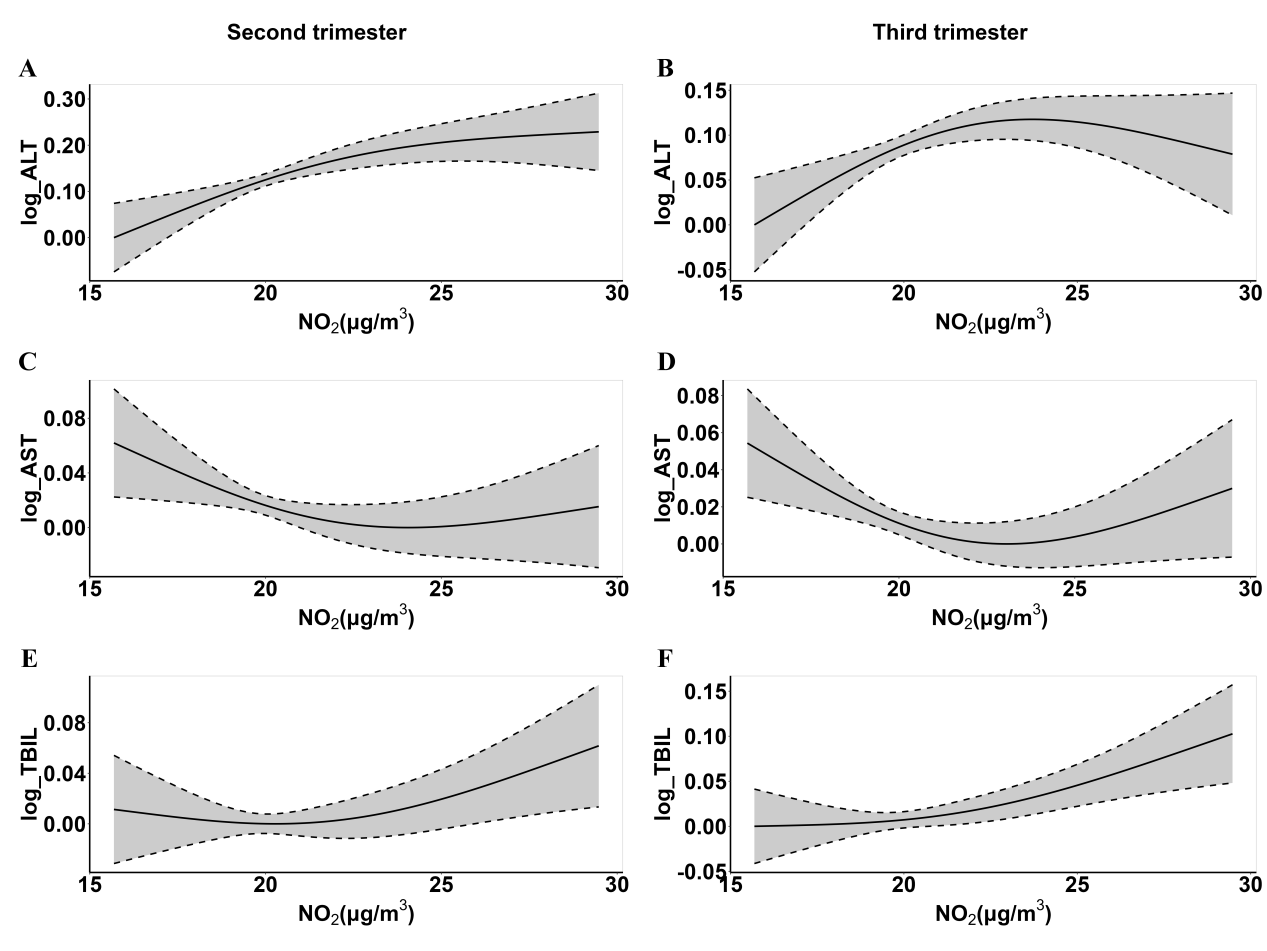


Fig. S2 Non-linear associations between NO_2_ air pollution exposure in the first trimester and maternal liver function in the second and third trimesters

NO_2_ air pollution exposure during the first trimester.

The lines show the log estimates and the shadowed areas represent the 95% confidence intervals.

Model adjusted for mean temperature and relative humidity using natural cubic splines (*df* = 3), exposure concentrations of O_3_, maternal age, gestational age at liver function testing, pregnancy methods, pre-pregnancy BMI, education, cigarette smoking, alcohol drinking, physical burden, gestational hypertension, gestational diabetes, status of HBsAg, taking medication during the first trimester, season at liver function testing.


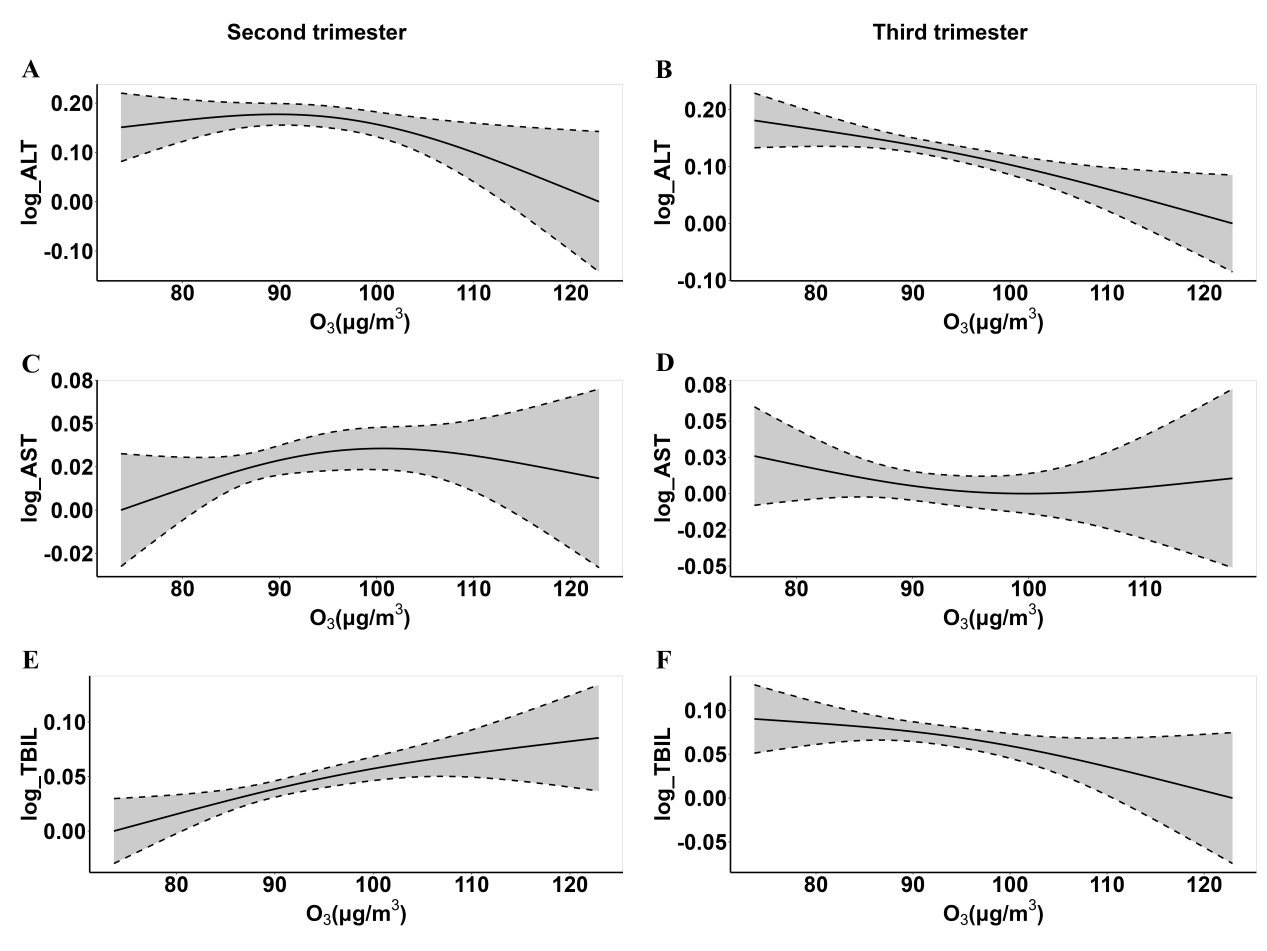
Fig. S3 Non-linear associations between O_3_ air pollution exposure in the first trimester and maternal liver function in the second and third trimesters

O_3_ air pollution exposure during the first trimester.

The lines show the log estimates and the shadowed areas represent the 95% confidence intervals.

Model adjusted for mean temperature and relative humidity using natural cubic splines (*df* = 3), exposure concentrations of NO_2_, maternal age, gestational age at liver function testing, pregnancy methods, pre-pregnancy BMI, education, cigarette smoking, alcohol drinking, physical burden, gestational hypertension, gestational diabetes, status of HBsAg, taking medication during the first trimester, season at liver function testing.


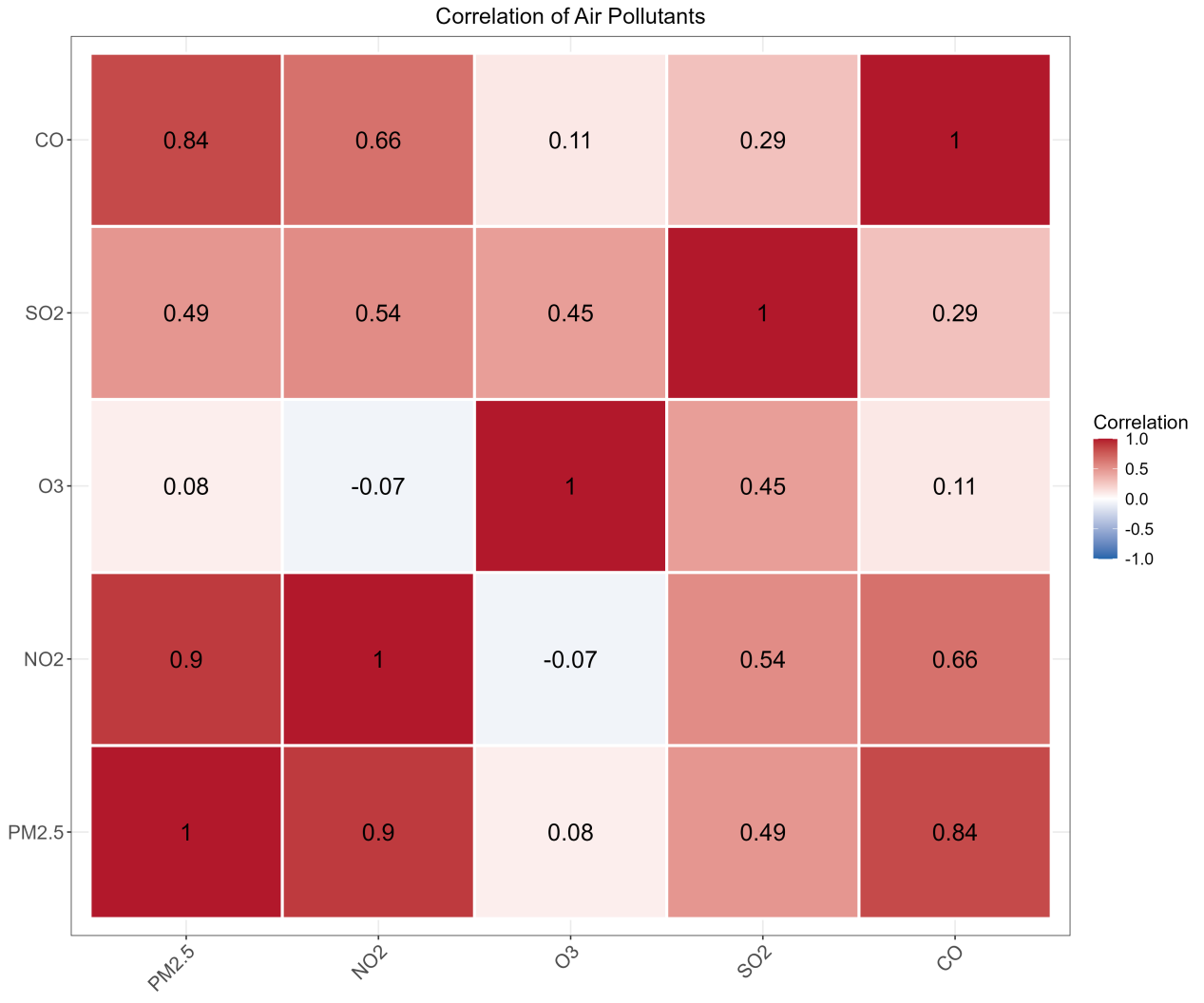


Fig. S4 Correlation of air pollutants

Exposure to air pollutants during the first trimester.


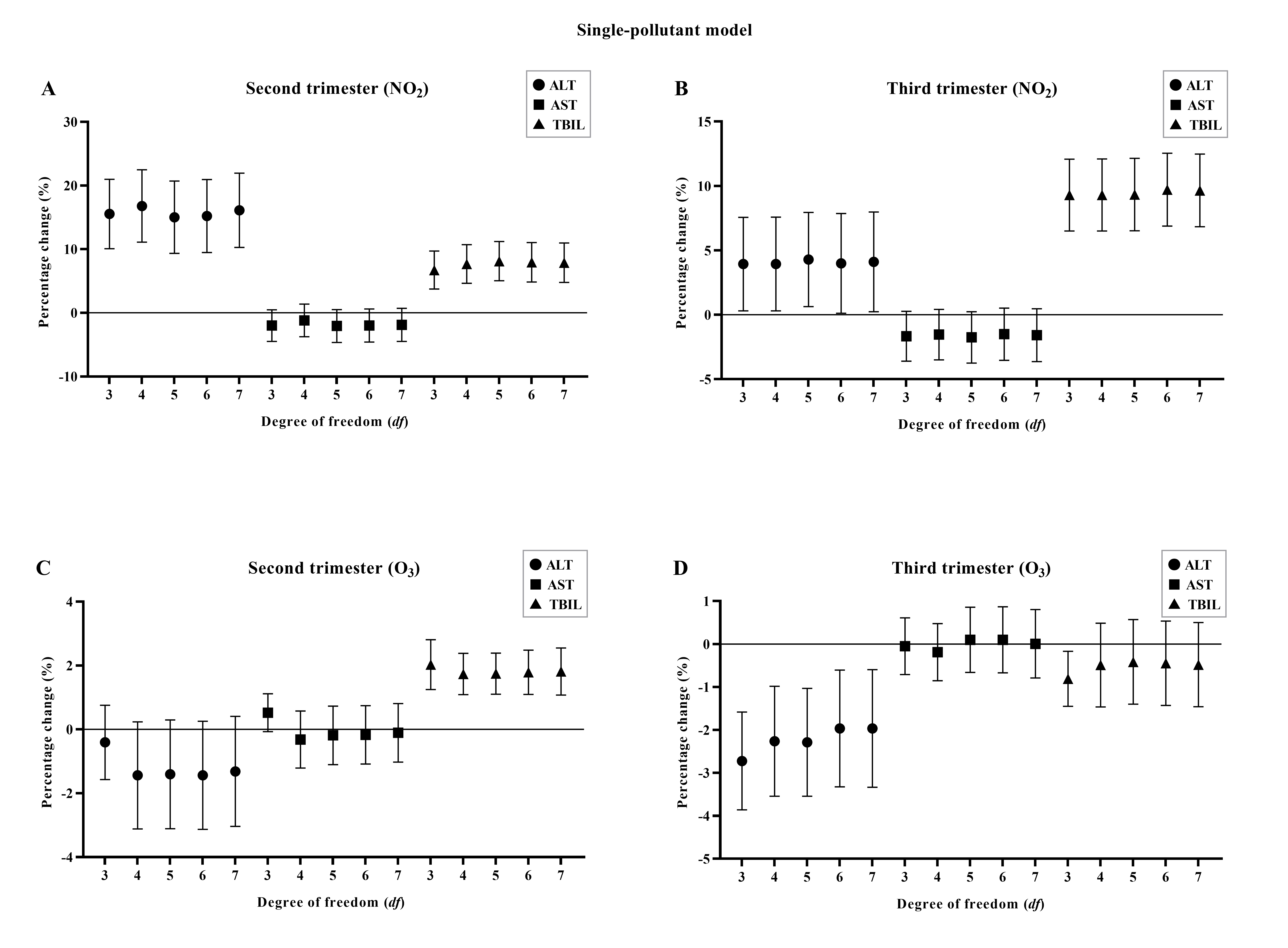


Fig. S5 Sensitivity analyses of associations of NO_2_ and O_3_ air pollution exposure in the first trimester with maternal liver function in the second and third trimesters by changing the degrees of freedom for the spline function from 4 to 7 in the single-pollutant model

NO_2_ and O_3_ air pollution exposure during the first trimester.

The symbols show the percentage change (%) and the upper and lower bars show the 95% confidence intervals.

Model adjusted for mean temperature and relative humidity using natural cubic splines (*df* = 3-7), maternal age, gestational age at liver function testing, pregnancy methods, pre-pregnancy BMI, education, cigarette smoking, alcohol drinking, physical burden, gestational hypertension, gestational diabetes, status of HBsAg, taking medication during the first trimester, season at liver function testing.


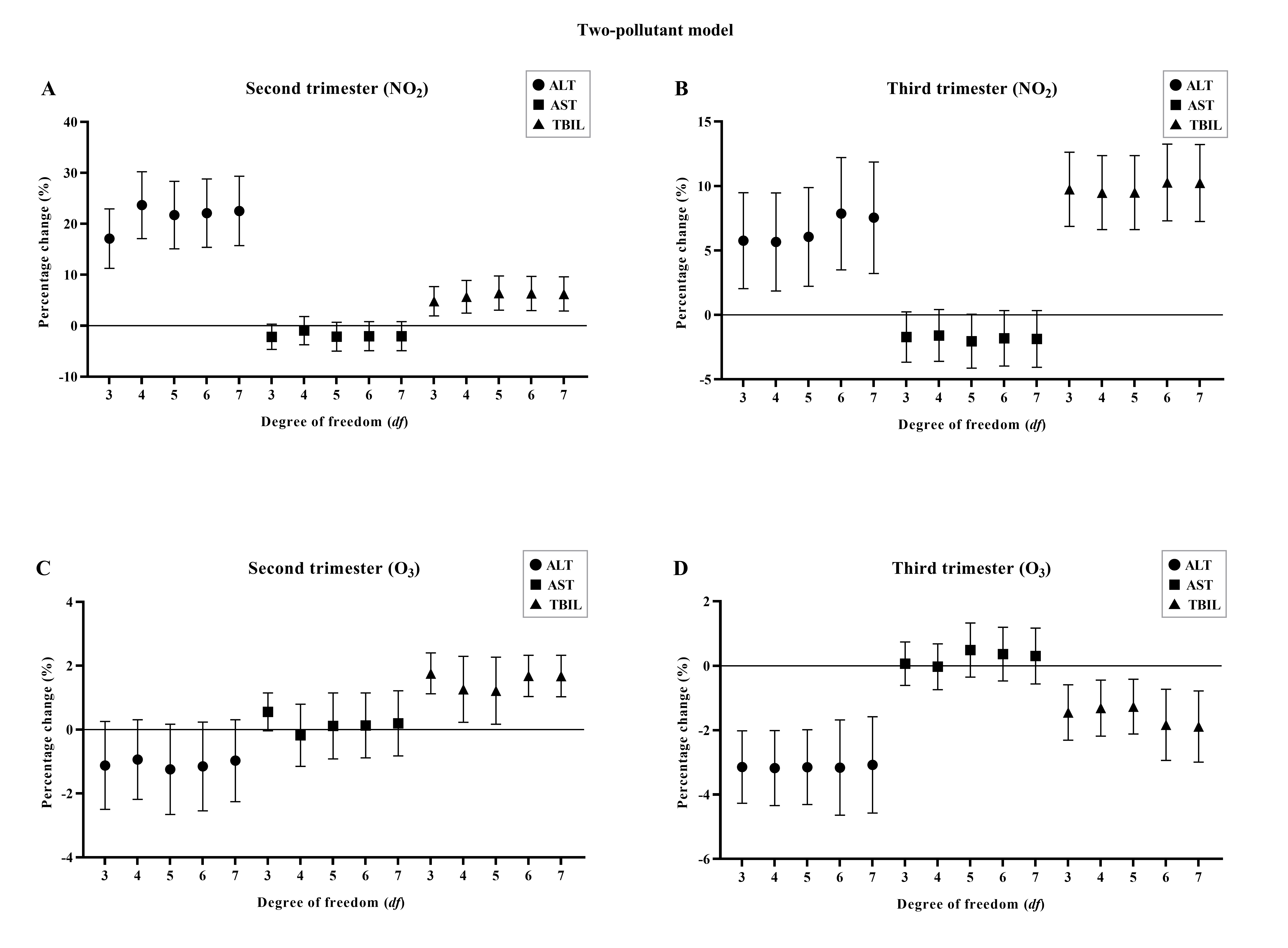


Fig. S6 Sensitivity analyses of associations of NO_2_ and O_3_ air pollution exposure in the first trimester with maternal liver function in the second and third trimesters by changing the degrees of freedom for the spline function from 4 to 7 in the two-pollutant model

NO_2_ and O_3_ air pollution exposure during the first trimester.

The symbols show the percentage change (%) and the upper and lower bars show the 95% confidence intervals.

Model adjusted for mean temperature and relative humidity using natural cubic splines (*df* = 3-7), exposure concentration of NO_2_ or O_3_, maternal age, gestational age at liver function testing, pregnancy methods, pre-pregnancy BMI, education, cigarette smoking, alcohol drinking, physical burden, gestational hypertension, gestational diabetes, status of HBsAg, taking medication during the first trimester, season at liver function testing.


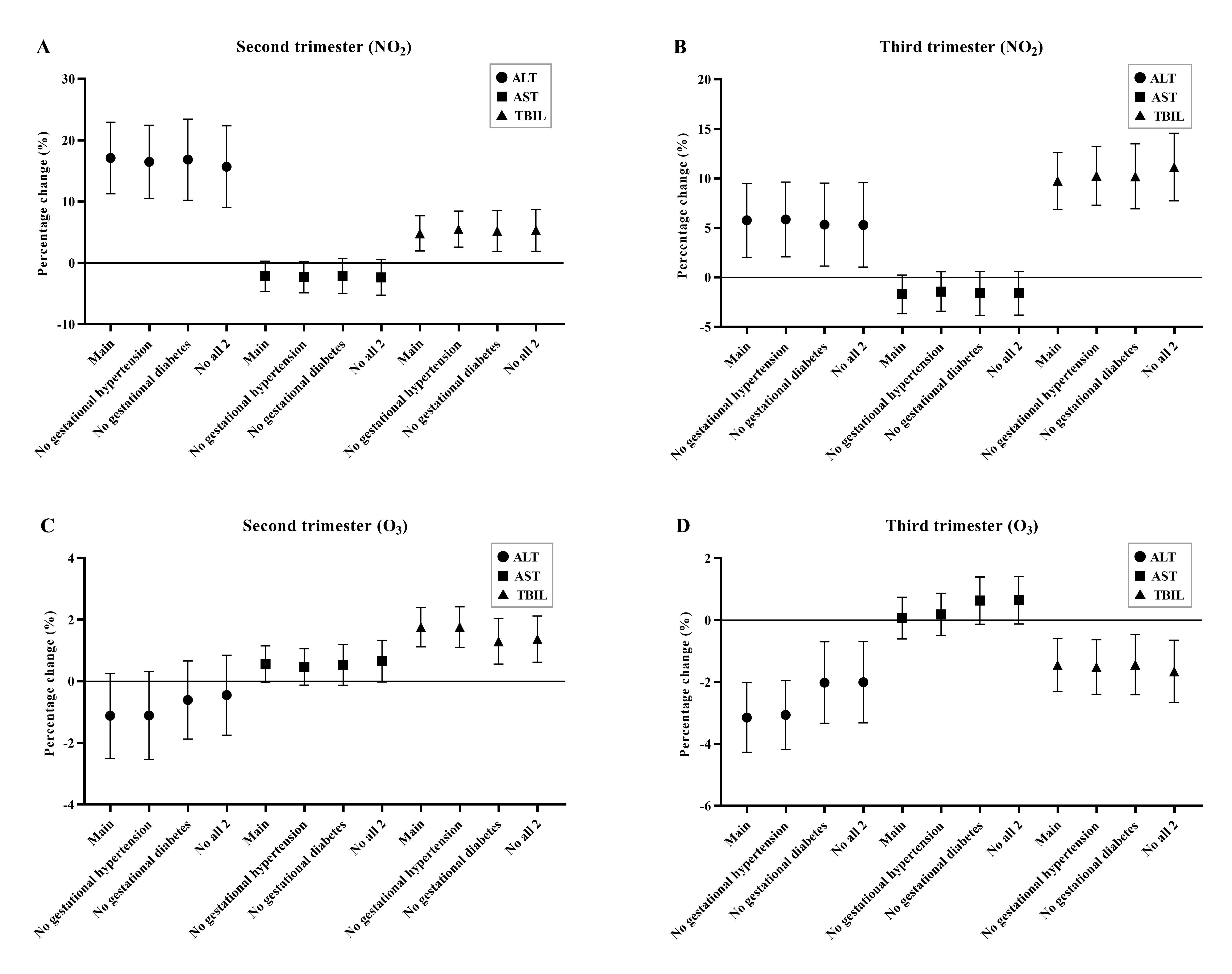


Fig. S7 Sensitivity analyses of associations of NO_2_ and O_3_ air pollution exposure in the first trimester with maternal liver function in the second and third trimesters by statuses of gestational hypertension and gestational diabetes

NO_2_ and O_3_ air pollution exposure during the first trimester.

The symbols show the percentage change (%) and the upper and lower bars show the 95% confidence intervals.

Model adjusted for mean temperature and relative humidity using natural cubic splines (*df* = 3), exposure concentration of NO_2_ or O_3_, maternal age, gestational age at liver function testing, pregnancy methods, pre-pregnancy BMI, education, cigarette smoking, alcohol drinking, physical burden, gestational hypertension, gestational diabetes, status of HBsAg, taking medication during the first trimester, season at liver function testing.
